# Molecular Mechanisms of Apoptosis Induction and Its Regulation by Fatty Acids in Pancreatic β-Cells

**DOI:** 10.3390/ijms22084285

**Published:** 2021-04-20

**Authors:** Jan Šrámek, Vlasta Němcová-Fürstová, Jan Kovář

**Affiliations:** Department of Biochemistry, Cell and Molecular Biology & Center for Research of Diabetes, Metabolism and Nutrition, Third Faculty of Medicine, Charles University, Ruská 87, 100 00 Prague, Czech Republic; jan.kovar@lf3.cuni.cz

**Keywords:** apoptosis induction, autophagy, ER stress, saturated fatty acid, unsaturated fatty acid, pancreatic β-cell, caspase, type 2 diabetes mellitus, kinase, fatty acid metabolism

## Abstract

Pancreatic β-cell failure and death contribute significantly to the pathogenesis of type 2 diabetes. One of the main factors responsible for β-cell dysfunction and subsequent cell death is chronic exposure to increased concentrations of FAs (fatty acids). The effect of FAs seems to depend particularly on the degree of their saturation. Saturated FAs induce apoptosis in pancreatic β-cells, whereas unsaturated FAs are well tolerated and are even capable of inhibiting the pro-apoptotic effect of saturated FAs. Molecular mechanisms of apoptosis induction by saturated FAs in β-cells are not completely elucidated. Saturated FAs induce ER stress, which in turn leads to activation of all ER stress pathways. When ER stress is severe or prolonged, apoptosis is induced. The main mediator seems to be the CHOP transcription factor. Via regulation of expression/activity of pro- and anti-apoptotic Bcl-2 family members, and potentially also through the increase in ROS production, CHOP switches on the mitochondrial pathway of apoptosis induction. ER stress signalling also possibly leads to autophagy signalling, which may activate caspase-8. Saturated FAs activate or inhibit various signalling pathways, i.e., p38 MAPK signalling, ERK signalling, ceramide signalling, Akt signalling and PKCδ signalling. This may lead to the activation of the mitochondrial pathway of apoptosis, as well. Particularly, the inhibition of the pro-survival Akt signalling seems to play an important role. This inhibition may be mediated by multiple pathways (e.g., ER stress signalling, PKCδ and ceramide) and could also consequence in autophagy signalling. Experimental evidence indicates the involvement of certain miRNAs in mechanisms of FA-induced β-cell apoptosis, as well. In the rather rare situations when unsaturated FAs are also shown to be pro-apoptotic, the mechanisms mediating this effect in β-cells seem to be the same as for saturated FAs. To conclude, FA-induced apoptosis rather appears to be preceded by complex cross talks of multiple signalling pathways. Some of these pathways may be regulated by decreased membrane fluidity due to saturated FA incorporation. Few data are available concerning molecular mechanisms mediating the protective effect of unsaturated FAs on the effect of saturated FAs. It seems that the main possible mechanism represents a rather inhibitory intervention into saturated FA-induced pro-apoptotic signalling than activation of some pro-survival signalling pathway(s) or metabolic interference in β-cells. This inhibitory intervention may be due to an increase of membrane fluidity.

## 1. Introduction

One of the main causes of T2DM (type 2 diabetes mellitus) is insufficient insulin production to cover the needs of the organism. Besides insulin resistance, this state results from the loss of β-cell function and viability. Elevated concentrations of FAs (fatty acids) in the blood are considered as one of the main factors responsible for this effect. The detrimental potential of FAs has been described in human as well as animal β-cells in vivo and in vitro [1,2,3,4,5,6,7,8,9]. There is convincing evidence that the toxicity of FAs depends particularly on the degree of their saturation. Saturated FAs (e.g., stearic and palmitic acid) induce apoptosis in pancreatic β-cells, whereas unsaturated FAs (e.g., oleic and palmitoleic acid) are usually well tolerated and are even capable of inhibiting the pro-apoptotic effect of saturated FAs [1,2,6,10,11,12,13,14,15].

Precise molecular mechanisms of apoptosis induction by saturated FAs and regulation of this induction by unsaturated FAs in β-cells remain unclear [16]. Nevertheless, it has been proposed that, e.g., pro-survival and pro-proliferative Akt and ERK (extracellular signal-regulated kinase) pathways, the p38 MAPK (mitogen-activated protein kinase) and PKC (protein kinase C) δ kinase stress pathways, ER (endoplasmic reticulum) stress and autophagy signalling could be involved. Concerning the mechanisms of apoptosis execution, the mitochondrial pathway of apoptosis induction and possibly also the death receptor pathway are engaged. The metabolism of FAs may also play a certain role here.

In this review, we deal with available data, obtained both in vitro and in vivo with β-cells of human as well as of rodent origin, and suggest possible mechanisms of FA-induced and FA-regulated apoptosis in pancreatic β-cells.

## 2. Effects of Fatty Acids on β-Cell Viability

### 2.1. Pro-Apoptotic Effect of Fatty Acids

The HFD (high-fat diet) leads to a decrease in the number of functional β-cells due to apoptosis in experimental animals [9,12,17,18,19,20]. These findings confirmed the existence of FA-induced apoptosis in β-cells in vivo and thus legitimize the comprehensive research of its molecular mechanisms in vitro.

The effect of various FA species on β-cells depends on their carbon chain length and the degree of saturation [14,21,22,23,24,25]. The predominant FAs in the blood serum are saturated PA (palmitic acid) and SA (stearic acid) and unsaturated POA (palmitoleic acid), OA (oleic acid) and LOA (linoleic acid) [26,27].

Saturated PA and SA cause the death of primary rat β-cells [28], as well as of β-cells in isolated rat [1,2,29] and human islets [1,4,10,30,31], and of β-cell lines [4,22,32,33,34]. SA seems to be more effective than PA in human β-cells and islets [10,14,25]. However, PA is usually tested as the representative saturated FA [1,4,30,31]. Interestingly, saturated FAs with a chain length shorter than 16 carbons, e.g., octanoate (8 carbons) and myristate (14 carbons), are ineffective as cell death inducers [6,35].

In contrast to PA and SA, unsaturated POA and OA have significantly lower or no pro-apoptotic effect on β-cells [6,10,11,14,22,36,37,38,39,40,41,42,43,44,45,46,47,48]. Interestingly, feeding of C57BL/6J mice with different diets also showed that HFD containing extra virgin olive oil, i.e., diet with high content of monounsaturated FAs, do not induce β-cell apoptosis, in contrast to lard-based HFD, i.e., diet with high content of saturated FAs [49]. Nevertheless, some studies showed the deleterious effect also in the case of unsaturated FAs [5,8,25,30,50,51,52,53,54,55,56,57]. Typically, this pro-apoptotic effect was less pronounced.

### 2.2. Inhibitory Effect of Unsaturated Fatty Acids on Pro-Apoptotic Effect of Saturated Fatty Acids

Although the evidence for the harmless effect of unsaturated FAs in β-cells is not fully unequivocal (see above), there are increasing experimental data showing the potential of unsaturated FAs to inhibit the detrimental effects of saturated FAs and saturated FA-induced cell death in the rat, as well as human, β-cells and islets [1,2,10,15,22,48,50,58,59,60,61].

Interestingly, monounsaturated FAs with a chain shorter than 16 carbons are completely ineffective [6] or significantly less effective in the inhibition of saturated FA-induced cell death in comparison with POA and OA [41,58]. Notably, the same pattern with respect to carbon chain length is seen for the toxicity of saturated FAs. Polyunsaturated FAs, e.g., LOA (C18:2) and γ-LOA (C18:3), or variants of OA (C18:1, n-9) where the double bond is located at different positions, e.g., vaccenic (C18:1, n-11) and petroselinic (C18:1, n-12) acid, were also protective [41,58]. Concerning the impact of *cis* and *trans* configuration on the inhibitory effect of unsaturated FAs, in rat BRIN-BD11 and INS-1 cells, palmitelaidic and EA (elaidic acid), were not as effective as their respective *cis* isomers POA and OA [24]. OA is protective against pristanic acid, a branched saturated FA, as well [41].

### 2.3. The Role of Fatty Acid Metabolism and Its Intermediates

#### 2.3.1. Fatty Acid Metabolism

The questions whether FAs are detrimental or protective, per se, or whether they act through some intermediates of their metabolism was answered by studies that compared the effect of FAs with the effect of their counterpart derivatives that cannot be metabolized by cells, e.g., alkyl esters and 2-bromo-derivatives of FAs [23]. Methyl esters of PA, where the formation of FA-CoA (coenzyme A) is blocked due to the unavailability of the carboxy group for esterification, are ineffective in apoptosis induction [41,54,62]. Similar data were obtained with bromo–palmitate, which can be esterified to CoA but cannot be oxidized further [22,39].

Surprisingly, methyl esters of unsaturated FAs are equipotent as cyto-protective agents, when compared with their non-methylated parental molecules [22,24,58]. They were also effective against apoptosis-inducing treatments other than saturated FAs [24]. In addition, a comparison of the *cis* and *trans* form of methyl-POA showed a similar difference in the inhibitory potential that was seen when non-methylated parental molecules were applied [24]. Thus, in marked contrast to saturated FAs, esterification of unsaturated FAs is not necessary for their anti-apoptotic effect.

Moreover, overexpression of CPT-1 (carnitine palmitoyl transferase 1) and the application of inhibitors of FA metabolism triacsin-C and etomoxir [4,22,37,59,63,64,65] confirmed that β-oxidation of FAs, per se, is not involved in the molecular mechanism by which FAs induce apoptosis. Rather, the glucose inhibition of fat oxidation and increased accumulation of acyl-CoA and/or metabolites derived from them in cytosol have pro-apoptotic consequences.

PA treatment leads to the activation of AMPK (AMP-activated protein kinase) [66]. AMPK is activated in response to low ATP [67] due to mitochondrial dysfunction caused by FAs in β-cells [16]. AMPK activation may represent a protective mechanism against excess intracellular fat storage via induction of fat oxidation and inhibition of lipogenesis. In line with this, the application of AMPK activators has a protective effect against PA-induced apoptosis [68]. The importance of β-oxidation as an anti-apoptotic mechanism against FA toxicity is supported by the finding that an increased capacity for the oxidation of FAs was documented in a PA-resistant subclone of murine MIN6 β-cells [36]. Metabolic conversion of saturated FAs to unsaturated FA species represents another potential protective mechanism. The protective role of SCD1 (stearoyl-CoA desaturase 1), which unsaturates PA and SA to POA and OA, was demonstrated in the rat, as well as human, β-cell lines [36,69,70]. Recently, the anti-apoptotic role of ELOVL2 (very long chain fatty acid elongase 2), whose major products are docosapentaenoic acid, against the deleterious effects of glucolipotoxicity in both rodent and human β-cells, was also shown [59].

#### 2.3.2. Triacylglycerols

FAs can be used for TAG (triacylglycerols) formation and stored in lipid droplets. Despite some contradictory evidence, this process appears to be rather protective [22,44,50,61,71,72]. Nevertheless, the composition of TAGs with respect to the content of saturated and unsaturated fatty acid chains is of importance, as well [44]. However, the role of TAG formation as a crucial determinant of β-cell viability under lipotoxic conditions is not probable, since unsaturated FAs are able to promote β-cell viability without affecting TAG formation and composition [22]. Interestingly, recent data indicate that, besides the deposition of TAGs into the lipid droplets, the lipid droplet-resident proteins (e.g., perilipin 2 and 5) are also involved in apoptosis regulation under lipotoxic conditions behind their role in the regulation of lipid storage [63,73].

#### 2.3.3. Ceramides

Increased supply of especially saturated FAs in β-cells also leads to ceramide production through de novo synthesis or by direct acylation of sphingosine [4,42,74,75,76]. The possible role of ceramide in FA-induced apoptosis was documented in β-cell lines as well as in isolated rat and human islets [1,2,3,65,75,77]. Importantly, the detrimental effect of ceramide on β-cell viability can be reproduced by exposure of islets to exogenous ceramide [1,2,65]. The incorporation of unsaturated FAs into ceramides is overall low. Nevertheless, it is noteworthy that markedly higher incorporation of *trans* FA species elaidate (18:1 *trans*-Δ9) and vaccenate (18:1 *trans*-Δ11) into ceramides and diglycerides, compared to OA, was shown in RINm5F cells [42]. This may explain the less beneficial effects of *trans* FAs, compared to their *cis* counterparts.

Ceramides can affect the activation of various cellular signalling pathways, including pathways leading to apoptosis induction [78]. For example, ceramides inhibit Akt [79,80] and activate JNK (c-Jun N-terminal kinase) [81], as well as ERK. In β-cells, the activity of Akt [37], JNK [54,82,83] and ERK is known to be altered by saturated FA treatment (see Section 4.1.1, Section 4.3.2 and Section 4.1.2). Ceramide signalling may also lead, via the activation of PP2A (protein phosphatase 2A) [84], to dephosphorylation of Bad (Bcl-2 associated death promoter) and Bcl-2 (B-cell lymphoma 2), which results in pro-apoptotic signalling [85].

Taken together, saturated FAs need to be esterified to FA-acyl-CoA to exert their deleterious effect. In contrast, unsaturated FAs do not need to be esterified to possess their anti-apoptotic activity. Various aspects of FA metabolism are involved in the pro- and anti-apoptotic effect of FAs, including FA oxidation, cellular conversion of saturated FAs into unsaturated species, channelling of FAs into TAG and lipid droplets, and ceramide formation. Notably, the formation of ceramide would well explain the difference in the toxicity of saturated and unsaturated FA species, as predominantly saturated FAs are substrates for ceramide production [86].

## 3. Initiation of Pro-Apoptotic Signalling by Fatty Acids

The real site where the FA-induced pro-apoptotic signalling originates is not revealed yet. It corresponds with the fact that the nature of the differential effect of saturated and unsaturated FAs on β-cell viability is unclear, as well. However, localisation of starting point(s) in the cytoplasm is easily believable, due to FA involvement in numerous metabolic and biosynthetic pathways (see previous sections).

Saturated FAs with rigid and straight acyl chain conformation decrease membrane fluidity after their incorporation into the lipid bilayers [87,88,89]. Saturated FA-induced pro-apoptotic signalling may, thus, originate at cell membranes, due to their increased rigidity, which, per se, may affect the activity of relevant membrane-associated proteins. This may, in turn, lead to signal transmission onto particular signalling pathway(s), which may result in a pro-apoptotic effect, and vice versa: increased membrane rigidity may also block activation of relevant membrane-associated proteins, leading to inhibition of particular pro-survival signalling pathway(s). Pro-survival Akt and ERK or pro-apoptotic p38 MAPK and PKCδ pathways, which are regulated by FAs in β-cells (see Section 4.1 and Section 4.2), may represent such signalling pathways. This hypothesis would be in line with the view that the development of T2DM is based on decreased membrane fluidity because of the excess of saturated FAs in the diet [90].

There is growing experimental evidence that saturated as well as unsaturated FAs are able to affect the activity of membrane proteins, e.g., PA activates TLR4 (Toll-like receptor 4) in hepatocellular carcinoma cells and epithelial rat kidney-derived cells [91,92]. Interestingly, in the latter cells, PA-induced TLR4 activation led to activation of c-Src and then to EGFR (epithelial growth factor receptor) activation within the c-Src/EGFR complex [92]. In endothelial cells, OA (but not PA) activates the EGFR and downstream signalling towards the ERK pathway independently of any autocrine secretion of EGF or other related mediators [93]. Such data indicate that besides the saturated FA-induced apoptotic signalling also anti-apoptotic signalling exerted by unsaturated FAs may originate already on the cell membrane (see Section 7).

The initiation point of FA-induced pro-apoptotic signalling may be also located in the ER (endoplasmic reticulum) where the excess of FAs or their metabolites disrupt its proper function and induce ER stress. Saturated FAs induce changes in ER membrane morphology [13,42], and ER membrane fluidity is very likely to be also affected. This may affect activation of ER membrane proteins IRE1α (inositol-requiring protein 1α), PERK (protein kinase RNA (PKR)-like ER kinase) and ATF6 (activating transcription factor 6) that are essentially involved in ER stress signalling (see Section 4.3). However, other membrane organelles or membrane-enclose structures, e.g., mitochondria or autophagosomes, may also represent the initiation point. Mitochondrial dysfunction due to FAs was documented in β-cells [16]. Saturated FA-induced decrease of mitochondrial membrane fluidity might play a role in this dysfunction, as well. Autophagosome fusion with lysosomes is necessary for proper autophagy functioning. More rigid autophagosomal membranes are less susceptible to fusion, which may lead to inhibition of autophagy flux and contribute to apoptosis induction.

However, modes of activation of the membrane and membrane-associated molecules by FAs from cytosol are likely, e.g., via the effect on their lipidation (i.e., myristoylation, palmitoylation, prenylation and glycosylphosphatidylinositol anchor), which in turn determine their membrane affinity, localization and function [94]. For example, saturated FAs were found to alter the membrane distribution of c-Src in fibroblasts via its myristoylation, which leads to subsequent JNK activation. Conversely, unsaturated FA prevented it [95].

We speculate that unsaturated FAs, with their bent tail, do not decrease but even increase membrane fluidity. The differential effects of saturated and unsaturated FAs on membrane-initiated cell signalling via membrane fluidity could thus well explain why saturated FAs can induce apoptosis in β-cells, while unsaturated FAs cannot. However, they do not provide a clue for the explanation of the pro-apoptotic effect of OA that is also observed in some studies [5,8,25,30,50,51,52,53,54,55,56,57].

GPRs (G protein-coupled receptors), membrane receptors for FAs, may also be supposed to switch on the FA-induced pro-apoptotic signalling, since some of them may regulate activation of various signalling pathways in β-cells, e.g., Akt, ERK, p38 MAPK or JNK, and GPR40 was shown to be possibly involved in FA-induced β-cell apoptosis (as well as all the mentioned kinases). However, its pro-survival role against saturated FA-induced apoptosis was also suggested in β-cells (see Section 4.5). Nevertheless, as FA receptors are activated by both saturated as well as unsaturated FAs, their activation would hardly explain the different pro-apoptotic properties of saturated and unsaturated FAs.

To conclude, the starting point(s) of the pathway leading to apoptosis induction by FAs in pancreatic β-cells is elusive so far. The localisation of this point(s) in the cytoplasm is easily conceivable, in respect to FA engagement into various metabolic and biosynthetic pathways. The initiation is likely to be mediated via some intermediate of FA metabolism, rather than FA molecule, per se, since the pro-apoptotic effect of saturated FAs is dependent on their esterification into FA-CoA [63,64]. However, the evidence concerning the effect of FAs, their intermediates and/or FA-modified molecules on membrane-associated signalling events is growing. Therefore, plasma and/or organelle membranes may also represent the initiation point(s).

## 4. Mechanisms Mediating Pro-Apoptotic Effects of Saturated Fatty Acids

Based on the experimental evidence available so far, FA-induced apoptosis seems to be preceded by complex cross talks of multiple signalling pathways with a differentially important contribution. These pathways are discussed below.

### 4.1. Pro-Survival and Pro-Proliferative Signalling

#### 4.1.1. Akt Pathway

The Akt pathway (PI3K (phosphoinositide 3-kinase)-PDK1 (phosphoinositide-dependent protein kinase 1)-Akt) is generally considered as the main pro-survival pathway among various cell types. It is activated by membrane RTKs (receptor tyrosine kinases) upon binding relevant ligand, usually referred to as survival factor [96]. Its pro-survival function was shown in human and rat pancreatic β-cells and islets, as well [97,98,99,100,101,102,103,104]. Most experimental evidence shows inhibition of the Akt pathway by saturated FAs [97,98,99,100,101,102]. In line with this, the overexpression of active Akt kinase increases β-cell size, mass and proliferation in islets of C57BL/6J mice [105,106]. Moreover, Akt signalling suppresses the pro-apoptotic JNK and/or p38 MAPK pathways in rat β-cells [107] and human islets [108].

Prominent downstream targets of active Akt are transcription factors from the FoxO (forkhead box O) family, i.e., FoxO1, 3a, 4 and 6. These proteins can mediate transcription of pro-apoptotic factors, e.g., multiple pro-apoptotic members of the Bcl-2 family and death receptor ligands, such as Fas ligand and TRAIL (tumour necrosis factor-related apoptosis-inducing ligand) [109]. However, when phosphorylated by Akt, FoxO proteins are held inactive in the cytoplasm and prevented from translocation into the nucleus (reviewed in [110]). FoxO1 is the most abundant isoform of the FoxO family in pancreatic β-cells [111], and inhibition of its transcriptional activity due to Akt phosphorylation and subsequent cytosolic localization was documented in β-cells [112,113]. In addition, the activation of FoxO1 and its translocation to the nucleus was proposed as a mediator of saturated FA-induced apoptosis in various β-cell lines [52,97,99,100,114,115,116], as well as in rodent islets [115,116,117]. Inhibition of FoxO1 activity is associated with pro-apoptotic Bax (Bcl-2-associated X) protein downregulation in rodent β-cell lines and murine islets [99,116]. Little is known about the role of the other FoxO proteins. FoxO3 is involved in PA-induced apoptosis of rat INS-1E β-cells via upregulation of pro-apoptotic proteins of the Bcl-2 family PUMA (the p53 upregulated modulator of apoptosis) and DP5 (death protein) [118].

Regarding the effect of unsaturated FAs on Akt activation, somewhat contradictory data are available [7,117,119,120,121]. Nevertheless, a decrease in Akt activity is commonly reported when the cell death-inducing effect of unsaturated FAs is evidenced [52,117,119]. Akt pathway activation was also suggested to mediate the inhibitory effect of LOA on the PA-induced apoptosis [62] (see Section 7).

Another well-known effect of Akt activation is inhibitory phosphorylation of Bad, a pro-apoptotic member of the Bcl-2 family. Akt also phosphorylates caspase-9 and GSK (glycogen synthase kinase) 3, which, e.g., inhibits Mcl-1 (myeloid cell leukemia-1), an anti-apoptotic member of the Bcl-2 protein family. Akt can also activate MDM2 (mouse double minute 2 homolog), which triggers p53 degradation (reviewed in [96]). This favours, besides other effects, the inhibition of the mitochondrial pathway of apoptosis activation. Surprisingly, the involvement of these general mechanisms of Akt signalling in FA-induced β-cell apoptosis has not been tested yet.

To summarize, the Akt pathway is inhibited by saturated FAs in β-cells. This leads to activation of FoxO1 and FoxO3 proteins and the upregulation and activation of pro-apoptotic members of the Bcl–2 family–H3–only and Bax proteins. This, in turn, facilitates activation of the mitochondrial pathway of apoptosis. Akt inhibition may also lead to activation of pro-apoptotic JNK and p38 MAPK. Activation of Akt by unsaturated FA may represent the mechanism of their inhibitory effect on saturated FA-induced apoptosis in β-cells.

#### 4.1.2. ERK Pathway

The ERK pathway (Raf-MEK (mitogen-activated protein kinase/ERK kinase)-ERK) is considered as the main pro-proliferative signalling pathway. Nevertheless, under some circumstances, ERK can function in a pro-apoptotic manner, as well. The ERK pathway, depending on cell type, is activated by cytokines/growth factors via the relevant RTKs. Subsequently, Ras GTPase is activated and transmits signalling further on Raf kinase [122,123]. In β-cells the ERK pathway can be activated by GPR40, as well [124].

The experimental evidence concerning the effect of saturated FAs on the ERK pathway in pancreatic β-cells is ambiguous. Both activation and inhibition of ERK was reported in the course of FA-induced apoptosis, and, confusingly, both ERK activation and inhibition were associated with pro-survival as well as pro-apoptotic effects [20,98,121,124,125,126,127,128,129,130,131].

The limited data concerning the effect of unsaturated FAs are also rather contradictory [83,120,127,132]. OA is able to inhibit the SA-induced inhibition of ERK in human NES2Y β-cells [131]. However, as selective inhibitors of ERK failed to influence the cyto-protective action of POA, the ERK pathway is unlikely to mediate the anti-apoptotic effect of unsaturated FAs [132].

Depending on the cell type, the ERK pathway activation is associated with the activation and the inhibition of both the death receptor (e.g., via caspase-8 regulation) and mitochondrial pathway (e.g., via Bad, Mcl-1 and caspase-9 regulation) of apoptosis induction [133,134]. However, data concerning the mechanisms downstream of the ERK pathway in the process of FA-induced β-cell apoptosis are missing. ERK is probably involved in the regulation of autophagy in β-cells [135].

Taken together, due to clear ambivalence between ERK activation and the effect of FAs on cell viability, the ERK pathway does not seem to play a decisive role in saturated FA-induced β-cell apoptosis nor in its inhibition by unsaturated FAs in pancreatic β-cells.

### 4.2. Stress Kinase Signalling

#### 4.2.1. p38 MAPK Pathway

The p38 MAPK (mitogen-activated protein kinase) pathway is one of the main cellular stress pathways. It is activated in response to various physical and chemical stresses. These factors activate GTP-binding proteins, (e.g., Rac1 (Ras-related C3 botulinum toxin substrate 1), Cdc42 (cell division control protein 42)), which subsequently phosphorylate MAP3Ks (e.g., ASK1 (apoptosis signal-regulating kinase), TAK1 (transforming growth factor-β-activated kinase 1), MLK (mixed-lineage kinase) 3). MAP3Ks then activate p38 MAPK [136]. In β-cells, p38 MAPK can be activated also by GPR40.

Saturated FAs activate the p38 MAPK pathway, and there is also consistent evidence for its involvement in pro-apoptotic signalling induced by saturated FAs in animal as well as human β-cells [114,125,131,137,138,139,140].

Sparse available data indicates no effect of OA application on p38 MAPK kinase activation. In addition, OA was shown to inhibit p38 MAPK activation following SA treatment [83,120]. However, when the pro-apoptotic effect of OA is detected, the activation of p38 MAPK is also found [114].

Concerning the downstream signalling of p38 MAPK in pancreatic β-cells, there is very little data available. In β-cells of diabetic db/db mice, p38 MAPK activation stimulates ER stress signalling and mediates β-cell apoptosis via decreased Bcl-2/Bax ratio [141]. In rat RINm5F β-cells, p38 MAPK is responsible for the activation of p53 during high glucose-induced apoptosis [142]. Activation of the p38 MAPK pathway by saturated FAs in human NES2Y β-cells leads to inhibition of the ERK pathway [131].

Summarizing, p38 MAPK activation is clearly associated with saturated FA-induced β-cell death potentially via the stimulation of the ER stress signalling and the inhibition of the ERK pathway. In line with this, the anti-apoptotic effect of unsaturated FAs seems to be linked with the inhibition of saturated FA-induced p38 MAPK activation.

#### 4.2.2. PKCδ Pathway

PKC isoforms are involved in various physiological responses, including apoptosis. PKCδ is the most studied isoform in the process of FA-induced β-cell apoptosis. It is activated by various stimuli by receptors coupled with the activation of PLC (phospho lipase C), as well as in manners independent of membrane receptors [143].

Saturated FAs activate PKCδ, and this kinase was shown to mediate FA-induced apoptosis in pancreatic β-cells [9,40,121,144]. Nuclear localization seems to be necessary for the pro-apoptotic function of PKCδ [40]. However, PKCδ was found to be dispensable for PA-induced apoptosis in rat BRIN-BD11 β-cells [145].

Unsaturated LOA, γ-LOA and arachidonic acid were documented to activate PKCδ in rat β-cells, while no effect of OA on PKCδ activation was found. Nevertheless, this may be due to the very low concentration of OA tested [121].

PKCδ can increase caspase-3 activation and regulate p53 activation in different cell types exposed to various pro-apoptotic stimuli. It can also activate JNK, ERK and p38 MAPK kinases and inhibit Akt (reviewed in [146]). Akt inhibition by PKCδ was also documented in β-cells [119].

To conclude, PKCδ seems to be involved in FA-induced β-cell apoptosis possibly via pro-survival Akt inhibition. However, the amount of available data is rather small.

### 4.3. Endoplasmic Reticulum Stress Signalling

Disturbances in the normal function of the ER lead to the UPR (unfolded protein response) and ER stress. This results in dissociation of the main ER chaperone BiP (immunoglobulin heavy chain-binding protein) from membrane proteins IRE1α, PERK and ATF6, leading to the activation of the respective pathways. Activated IRE1α causes unconventional splicing of mRNA for XBP1 (X-box binding protein 1), which leads to the translation of the active transcription factor (XBP1s). The second branch downstream from IRE1α represents JNK activation. Activation of the PERK results in the eIF2α (eukaryotic initiation factor 2α) phosphorylation. ATF6 pathway activation leads to translocation of ATF6 transcription factor to the nucleus. Upon activation, these signalling pathways primarily aim at the restoration of ER homeostasis, e.g., by increased expression of chaperones, such as BiP [147]. If ER dysfunction is severe or prolonged, signalling leading to apoptosis induction is activated by mechanisms that are still not completely clear. Proposed mediators are transcription factor CHOP (CCAAT-enhancer-binding protein (C/EBP) homologous protein) [148] and possibly also activation of caspase-4/-12 [149].

The role of ER stress in type 2 diabetes pathogenesis was indicated by increased expression of ER stress markers BiP and CHOP in islets from type 2 diabetes patients [38,150]. Furthermore, an increased expression of phospho-eIF2α, ATF4 (activating transcription factor 4) and CHOP mRNA was shown in islets of diabetic db/db mice [38,151].

Treatment of β-cells with saturated FAs results in remarkable changes in cell membrane morphology, including the ER membrane [8,13,29,30,42,44,152]. On the contrary, *cis* and *trans* unsaturated FAs, as well as co-application of saturated FAs with unsaturated FAs, induce only weak or no changes in ER morphology [13,29,42]. In parallel with the pro- and anti-apoptotic effect of FAs, the conversion of saturated FAs into FA-CoA is necessary for ER stress induction [54,58]. However, no metabolic conversion of unsaturated FAs is needed for the inhibitory effect on ER stress signalling activated by saturated FAs [13,58] (see also Section 2.3).

Nevertheless, how FAs application leads to ER stress is unclear. FAs are known to affect plasma membrane fluidity after their incorporation into the lipid bilayers [87,88,89] and may affect the activity of some membrane proteins [91,92,93]. We hypothesize that the activation of ER stress membrane sensors may also be induced by the effect of FAs on ER membrane fluidity. Moreover, an accumulation of saturated FAs in the ER was shown in β-cells [153], so it may be also reasonable to speculate that the presence of excess FAs in the ER could somehow interrupt the proper function of ER, e.g., protein folding, which would result in ER stress signalling.

#### 4.3.1. PERK Pathway

Saturated FAs activate PERK, and this pathway was convincingly demonstrated to be involved in the process of FA-induced apoptosis in rodent as well as human β-cells and human islets [5,13,28,29,31,38,54,83,117,154,155,156,157,158,159,160]. Surprisingly, in contrast to other cell types where sustained phosphorylation of eIF2α protects against ER stress, in β-cells, it was found to be pro-apoptotic [28,31].

On the other hand, PERK pathway is not activated by unsaturated FAs [8,13,28,29,31,46,54,83,154,158], unless their application is associated with apoptosis induction [5,159]. Moreover, an inhibitory effect of OA (or POA) on PA-induced PERK pathway activation was documented in rodent β-cells [13,29,46].

Mechanisms of saturated FA-induced β-cell apoptosis downstream of the PERK pathway activation include, besides induction of CHOP expression, downregulation of several anti-apoptotic proteins of Bcl-2 family, e.g., Mcl-1 [161], Bcl-2 [118,162], Bcl-xL (B-cell lymphoma-extra large) [118] and Bcl-W [162], as well as upregulation of the pro-apoptotic BH-3 only protein DP5 [118]. The effect on Bcl-2 and Bcl-W is mediated by decreased p53 activation and increased miR34a-5p expression [162]. The PERK pathway can also activate autophagy in β-cells [163,164], but conflicting data exist concerning PERK-mediated Akt regulation [118,165].

#### 4.3.2. IRE1α Pathway

Based on the detection of XBP1 splicing and IRE1α phosphorylation, there is clear evidence that saturated FAs activate the IRE1α-XBP1s branch of IRE1α signalling in rodent as well as human β-cells and human islets [5,8,15,28,29,38,46,54,82,83,155,157,160]. In contrast, OA application leads to no, minimal or only transient XBP1 splicing [5,8,28,29,38,46,54,83]. Moreover, OA is able to inhibit saturated FA-induced XBP1 silencing [29,54,83]. Its sustained production is associated with β-cell apoptosis [166]. Surprisingly, in response to PA application, XBP1s may activate Akt [103] and inhibit FoxO1 in β-cells [167].

Saturated FAs activate the IRE1α-JNK-c-Jun branch of ER stress signalling, as well [54,82,98,118,138,139,155,168,169,170,171,172,173], while unsaturated FAs, rather, have no effect on JNK activation [54,83,114,120]. OA is even able to inhibit SA-induced activation of JNK in human β-cells [83]. JNK activation was also suggested to regulate the transition from adaptive to apoptotic UPR [169] and, thus, plays an important role in the switch from the pro-survival ER stress signalling to activation of apoptotic pathways. JNK upregulates pro-apoptotic proteins DP5 and PUMA [118,174], and induces autophagy [175] in PA-treated β-cells. After various non-lipid apoptotic stimuli, this kinase was shown to inhibit insulin-induced Akt phosphorylation [176] and to increase p53 expression [177] in β-cells.

Nevertheless, the role of JNK in FA-induced β-cell apoptosis remains to be elucidated. JNK inhibition resulted in partial reduction of PA-induced apoptosis in rat β-cells [54] and reduced cell death in islets from diabetic db/db mice [169]. However, JNK inhibition was not protective in SA-treated human β-cells [82], and JNK1 knock-down (but not JNK2 and JNK3) increased PA-induced apoptosis in rat β-cells, indicating even a pro-survival role [174]. In addition, it must be noted that mechanisms of JNK activation and regulation independent of ER stress signalling, e.g., activation downstream of TLR4 [178], can be included in FA-induced β-cell apoptosis, as well.

#### 4.3.3. ATF6 Pathway

Concerning the ATF6 pathway, less unequivocal experimental data exist. Activation by saturated FAs was reported in human [82,179] as well as rodent β-cells [5,38,180]. However, the results of some other studies [28,54] are hard to interpret, due to indirect and less specific methods of analysis employed. Rather, no effect of OA on ATF6 activation was reported (directly or indirectly) in rodent [38] nor human β-cells [82,179]. In human β-cells, OA was even reported to block SA-induced ATF6 cleavage [179]. Nevertheless, some basal expression of active ATF6 (ATF6α-p50) was shown to be necessary for β-cell survival, even under non-stress conditions, and a pro-survival role of ATF6 was documented by others, as well [181,182]. A key mediator of ATF6-induced pro-apoptotic signalling is transcription factor CHOP [151]. This pathway also can regulate the activation of p38 MAPK and JNK in β-cells [181].

#### 4.3.4. Mediator and Effector Molecules of Endoplasmic Reticulum Stress-Induced Signalling

All branches of ER stress signalling converge into the induction of chaperone expression in order to potentiate the ER folding capacity. The most prominent chaperone induced by ER stress is BiP and is commonly detected as an ER stress marker.

Experimental evidence concerning the effect of saturated and unsaturated FA treatment on BiP expression in β-cells is not entirely concise. Mostly, increased BiP protein or mRNA levels in response to saturated FAs were reported in rodent [5,15,28,38,83,154,156,180,183] as well as human β-cells [15,83] and islets [31,54,156]. However, no induction of BiP expression due to saturated FAs was observed [8,13,30,46]. Concerning unsaturated FAs, similar ambiguity exists. Induction of BiP expression was not found in several studies [8,13,31,38,54,82,83,156] but detected in others [5,28,46,154]. In addition, OA was reported to inhibit saturated FA-increased expression of BiP in human β-cells [5,28,46,82,83,154]. Importantly, overexpression of BiP was found protective against PA-induced apoptosis in murine MIN6 β-cells [38]; however, it was not confirmed in rat INS-1 β-cells [30].

The available experimental evidence concerning mechanisms that directly link FA-induced ER stress with apoptosis induction in β-cells is not very extensive and includes mostly caspase-12 and CHOP as possible mediators. Inflammatory caspase-12 is a known effector molecule of ER stress-induced cell death in rodents [149]. Its activation following PA treatment was demonstrated in rat β-cells [54,130,180]. As far as we know, there is no evidence available concerning caspase-4 activation due to ER stress in β-cells yet. Nevertheless, caspase-4 activation following PA treatment is likely, since it was shown in PA-treated human hepatocytes [184].

As to the role of CHOP, in rodent (e.g., [5,13,32,38,113,157,158,180,185]) and human [83] β-cells, rat primary β-cells [54], and in human islets [31,54,158], CHOP expression is increased by saturated FAs but not by unsaturated FAs. These are even able to inhibit this effect of saturated FAs. More importantly, CHOP deletion improves β-cell function and promotes cell survival in multiple mouse models of diabetes [186]. siRNA-mediated knock-down of CHOP only delayed but did not inhibit the PA-induced apoptosis in rat β-cells, suggesting other molecules to be involved in apoptosis induction downstream of ER stress, as well [54]. It must be also noted that the expression of CHOP does not always necessarily correlate with the pro-apoptotic effect of FAs nor the progress of apoptosis induction [5,8,28].

Concerning the FA-induced pro-apoptotic signalling downstream of CHOP in β-cells, CHOP regulates the expression or localization of various proteins of the Bcl-2 family, e.g., pro-apoptotic Bax, Bim, Bcl-xL and anti-apoptotic Bcl-2, as well as of death receptor 5 [187]. In various cell types, CHOP can increase the production of ROS (reactive oxygen species) [188], leading to ER calcium release [189]. ROS induction by CHOP was also found in β-cells [186]. CHOP also suppresses p21 in β-cells and, thus, the switch from the pro-survival to the pro-apoptotic response of ER stress [190]. In high glucose-treated β-cells, CHOP was also documented to regulate PUMA independently of Akt-FoxO3 signalling [191].

The available data show that all three ER stress pathways (PERK, IRE1α and ATF6) are activated due to saturated FAs and that these pathways are more or less crucially involved in FA-induced apoptosis of β-cells. Unsaturated FAs do not activate ER stress pathways in the case of their non-apoptotic effect. They are even able to inhibit saturated FA-induced activation of these pathways. ER stress pathways may lead to the activation of autophagy in β-cells. The expression of ER chaperone BiP is increased due to saturated FA application as a defensive response of β-cells. CHOP and possibly also caspase-12 represents the most important, but certainly not the sole mediators, of FA-induced apoptosis downstream of ER stress. CHOP mediates FA-induced apoptosis mainly via the regulation of the Bcl-2 family and possibly also via the increase of ROS production.

### 4.4. Autophagy Signalling

Macroautophagy, commonly referred to as autophagy, is responsible for the removal of defective and damaged cellular components. However, under nutrition stress, removal of non-damaged cellular components may be also initiated in a cell. Autophagosomes engulf cytoplasmic components, including cytosolic proteins and organelles. Subsequently, they fuse with lysosomes, and the internal material is degraded. Rather complex cross talks of various cellular signalling pathways precede autophagy activation. This includes ERK, Akt and ER stress signalling; AMPK and the main autophagy regulator mTOR [192,193,194,195,196]. Akt activates mTOR and thus inhibits autophagy in β-cells [197]. Nevertheless, available data concerning the involvement of mTOR in autophagy activation obtained on β-cells are rather rare [163,198]. Interestingly, HFD was shown to activate mTOR in murine islets [175,199,200,201].

The proper functioning of β-cells is dependent on a functional autophagy pathway [202,203]. It was evidenced on rodent models [204,205,206] as well as on T2DM patients [207,208] that defects in the autophagy pathway are associated with T2DM. Furthermore, increased autophagosome formation was found in ZDF (Zucker diabetic fatty) rats, db/db mice and HFD-fed C57BL/6 mice, as well as in FA-treated rat β-cells [201,202,209]. However, whether saturated FA application accelerates or impairs the autophagy flux is ambiguous [152,175,208,210]. The deleterious effect of saturated FAs within autophagy seems to be exerted via a reduction in lysosomal pH and impairment of autophagosome–lysosome fusion [210]. This may result in apoptosis triggering, probably via the formation of so-called iDISC, an intracellular form of DISC (death-inducing signalling complex), and subsequent caspase-8 activation [193]. Unsaturated FAs antagonize the effect of saturated FAs on autophagy [211,212].

Nevertheless, it seems that autophagy activated in response to FA application represents rather a cyto-protective mechanism, as demonstrated in several β-cells lines and human islets [42,164,200,201,202,213,214]. However, when the effect of FAs is severe or prolonged, FAs block autophagy flux, perhaps due to the impairment of autophagosome-lysosome fusion. Unsaturated FAs are able to inhibit the saturated FA-induced autophagy, since they inhibit the negative effects of saturated FAs on the autophagy flux as well as saturated FA-induced ER stress.

### 4.5. Other Mechanisms Involved

Besides the above discussed mechanisms, i.e., pro-survival and pro-proliferative signalling, stress signalling and autophagy signalling, some other mechanisms were suggested to be engaged in FA-induced apoptosis.

Saturated FAs treatment is associated with an increase in ROS in rodent β-cell lines [41,53,98,185,214,215,216,217,218,219,220] (not in a human β-cell line [15]), and ROS increase was found to be involved in PA-induced apoptosis in rat INS-1 β-cells [221]. OA treatment causes this effect only in experimental models, where it also results in cell death induction [53,56,222,223]. In rat RINm5F β-cells, OA even prevented PA-induced ROS generation [41], and a protective effect of OA against mitochondrial membrane permeabilization by long term PA exposure in murine MIN6 β-cells was also shown [224]. The ROS increase is associated with translocation of pro-apoptotic Bcl-2 proteins (e.g., Bax and Bad) from the cytosol to mitochondria and subsequent cytochrome c release. This effect of ROS on Bcl-2 proteins may be mediated by p53, JNK or ER stress [225]. In β-cells, mechanisms of the pro-apoptotic effect of increased ROS were not systematically studied. However, ROS were demonstrated to regulate PA-induced ER stress [221].

GPR40 (G protein-coupled receptor 40), a plasma membrane receptor for FAs, is selectively expressed on β-cells and is activated by medium- and long-chain FAs. It is suggested to mediate the acute as well as the chronic effect of FAs on β-cell function [226]. GPR40 knock-out protects mice from obesity induced by HFD and overexpression of this molecule leads to diabetes [226]. However, experiments employing β-cell lines bring rather ambiguous data [120,124,138]. Interestingly, GPR40 was suggested to be involved in the anti-apoptotic effect of unsaturated FAs against saturated FA-induced β-cell apoptosis [120]. This receptor was documented to activate ERK, p38 MAPK and Akt kinases in β-cells [124,138,227].

p53 activation by FAs during cell-death induction was demonstrated in animal β-cell lines and animal and human islets [52,114,162,228,229]. This molecule is generally considered to regulate the expression/activation of Bax-like, BH3-only and Bcl-2 proteins. For example, p53 was shown to downregulate Bcl-2 and Bcl-W proteins via miR-34a-5p in β-cells of C57BL/6 mice on HFD [162]. However, the direct connection between p53 and Bax-like proteins was not systematically studied in FA-treated β-cells. The upregulation of proteins of the Bcl-2 family can occur in a p53-independent mode, as well, e.g., the upregulation of BH3-only protein PUMA by PA in INS-1 β-cells [118].

An increasing amount of data also supports the importance of miRNAs in the regulation of FA-induced β-cell apoptosis. Possible involvement was already shown for miR24 [230], miR34a [229,231,232], miR146 [229], miR-375 [233], miR-182-5p [234] and miR-34a-5p [162], as well as for miR-297b-5p and miR-374c-5p [235,236], in rodent β-cells and islets. Overexpression miR-297b-5p and mir24 were even shown protective against FA-induced apoptosis [230,235]. Some molecular mechanisms of miRNAs were already revealed during lipotoxicity in β-cells: e.g., miR-34a-5p was found to downregulate anti-apoptotic Bcl-2 and Bcl-W proteins [231], while miR-21 was able to inhibit XBP1 production [230], PERK pathway and p38 MAPK [237].

The surplus of NO (nitric oxide) produced by the iNOS (an inducible form of NO synthase (NOS)) is an established mediator of β-cell apoptosis [238] and was speculated to be involved in apoptosis induction by FAs, as well. However, the available data are contradictory [3,6,43,50,65,239,240]. Interestingly, upregulation of the neuronal form of NOS by PA in rat β-cells was also shown, and its downregulation increased PA-induced apoptosis [239].

Calcium release from the ER is also known to be involved in the regulation of β-cell viability by FAs [37,241,242,243,244]. Calcium-dependent cysteine protease calpain-2, activated in response to intracellular calcium overload, is required for PA-induced expression of CHOP and, interestingly, also for the activation of caspase-12 and caspase-3 in β-TC3 β-cells [180].

Other molecules/events speculated to play a role in the mechanisms of FA-induced β-cell apoptosis include activation of transcription factor NF-κB (nuclear factor kappa B) [5,104,114,245,246], GPR120 [139], FAT/CD36 (fatty acid translocase/cluster of differentiation 36) [247], cathepsin D [208] and calpain-10 [248]; degradation of carboxypeptidase E [39]; cellular localization of annexin A2 and A4, reticulocalbin-2 and 14-3-3γ [249]; expression of Nck1 [250] and JunB downregulation [103].

Taken together, the mechanisms of FA-induced apoptosis are not completely elucidated, and the number of other molecules or events found to be also engaged is growing. It seems quite clearly that the increase of ROS production, which may be in β-cell associated with ER stress signalling, as well as p53 activation is involved. Concerning the role of others, i.e., changed expression of certain miRNAs; calcium release from ER, GPR40 and NF-κB activation; and iNOS-related NO production, there are rather few and often contradictory data available.

## 5. Execution of Apoptosis Induction by Fatty Acids

Concerning apoptosis induction by FAs in pancreatic β-cells, there is experimental evidence for the involvement of both the mitochondrial as well as the death receptor pathway of apoptosis induction. Both of these pathways are discussed below.

### 5.1. Mitochondrial Pathway of Apoptosis Induction

When this pathway, also known as the intrinsic pathway, is triggered, cytochrome c is released from mitochondrial intermembrane space into cytosol enabling assembly of a multiprotein complex called apoptosome that functions as an activating platform for initiator caspase-9 [251]. Cytochrome c release is controlled at the level of mitochondrial membrane permeability by proteins of the Bcl-2 family.

Members of this family of proteins are divided into the pro- and anti-apoptotic group, according to their action in the regulation of apoptosis induction. Pro-apoptotic members are distinguished into two subfamilies based on their structure in respect to the content of so-called BH (Bcl-2 homology) domains: (1) the Bax-like proteins and (2) the BH3-only proteins. The Bax-like proteins, e.g., Bax and Bak, are able to form channels in the outer mitochondrial membrane and thus enable cytochrome c release. Under normal conditions, Bax-like proteins are sequestered in an inactive state by binding to anti-apoptotic proteins of the Bcl-2 subfamily, e.g., Bcl-2, Mcl-1 and Bcl-xL. BH3-only proteins, e.g., Bid, Bad, Bim and Puma, contain only one BH domain (BH3) and function via forming complexes with anti-apoptotic proteins of the Bcl-2 subfamily, thus liberating the Bax-like proteins to form channels in the mitochondrial membrane [252].

The exact mechanisms, how the expression/activity of proteins of the Bcl-2 family is regulated during apoptosis induction by FAs in β-cells, have not been explicitly addressed so far. However, a lot of signalling pathways regulated by FA application are known to result in changed expression or activation of proteins of the Bcl-2 family and some of these events, i.e., Akt pathway, ceramide signalling, CHOP, JNK and p53, were demonstrated in β-cells, as well (see the respective previous sections).

Saturated FA treatment tends commonly to increase the level of Bax-like proteins Bax [53,100,102,113,130,219,253] and Bak [254] in islets and β-cell lines. Bax translocation from the cytosol to the mitochondria was also evidenced [118]. More importantly, Bax expression is increased in pancreatic sections from patients with T2DM, compared to non-diabetic controls [38]. In addition, an increased Bax/Bcl-2 ratio, indicating higher susceptibility to apoptosis, was found in the pancreatic tissue of HFD-fed mice [255].

Saturated FAs also increase the expression of the level of BH3-only subfamily proteins PUMA [102,118,256], DP5 [118] and Bim [257] in islets and β-cell lines, but no effect of PA on Bim expression [118,161] and Bad phosphorylation [258] (leading to its activation) was found. The role of Puma and DP5 in FA-induced β-cell apoptosis is very likely, since their knock-down decreased PA-induced apoptosis in the rat as well as primary rat β-cells and human islets [118].

Regarding the effect of saturated FAs on the expression of proteins of the Bcl-2 subfamily in β-cells, the level of Bcl-2 [1,37,53,100,219,254], Bcl-xL [37,102,254], Bcl-W [162] and Mcl-1 [102,254] stayed rather unchanged or decreased during PA or SA treatment in rodent cell lines or rat and human islets. Moreover, Bcl-2 and Bcl-xL knock-down enhanced PA-induced apoptosis [118].

Only a few data exist concerning the effect of unsaturated FAs on the expression/activity of proteins of the Bcl-2 family. POA and OA application increased the expression of the Bcl-2 protein in human islets [1] and were even able to revert the PA-induced decrease in Bcl-2 expression in human islets, as well as in rat β-cells [1,29].

The Bax-like proteins are able to form channels in the outer mitochondrial membrane and thus enable cytochrome c release. The release of cytochrome c from mitochondria was mostly detected after saturated FAs treatment in β-cell lines and islets [1,2,53,259], while no release of cytochrome c occured due to unsaturated FAs application [1,2]. Moreover, POA and OA co-application even blocked cytochrome c release induced by PA [1,2].

Released cytochrome c is essential for subsequent activation of caspase-9 within the apoptosome complex. However, caspase-9 can also be activated by caspase-12 [260]. Caspase-9 activation was documented in rodent β-cells after PA application [41,118,130,174,261], as well as after unsaturated OA and LOA application, in experimental models, where they exhibited a pro-apoptotic rather than anti-apoptotic effect [52,56,262]. In contrast, OA was shown to inhibit SA-induced caspase-9 activation and cell death in human β-cells [15,83].

Taken together, there is clear evidence that saturated FAs activate the mitochondrial pathway of apoptosis induction in β-cells via increased expression/activity of pro-apoptotic proteins of the Bcl-2 family and/or decreased expression/activity of anti-apoptotic proteins of the Bcl-2 family. It leads to cytochrome c release and, subsequently, to caspase-9 activation.

### 5.2. Death Receptor Pathway of Apoptosis Induction

The death receptor pathway is triggered via cell surface receptors called death receptors, e.g., receptors for TNF-α (tumour necrosis factor-α), FasL (Fas ligand) and TRAIL (tumour necrosis factor-α-related apoptosis-inducing ligand). The binding of these receptors with their respective ligands, called death ligands, enables the assembly of the multiprotein complex called DISC. Here, initiator caspase-8 or -10 become activated, and, thus, they can activate by cleavage the executioner caspases [251]. Active caspase-8 or -10 can also amplify apoptosis induction by engagement of the mitochondrial pathway via cleavage of Bid, BH3-only protein of Bcl-2 family, to tBid. tBid promotes the oligomerization/insertion of Bax into the mitochondrial outer membrane and the release of cytochrome c [251].

PA and SA treatment activates caspase-8 in human [15,82,83,131] and rodent β-cell lines [41,261] while OA co-application inhibits it [15,41]. Importantly, increased caspase-8 activation was shown in islets from T2DM patients, compared to non-diabetic controls, as well [263]. HFD-fed mice were found to express more caspase-8 [255] and, interestingly, HFD-fed mice lacking caspase-8 lost less β-cell mass and were protected from the development of T2DM [264].

However, the mechanism of death receptor pathway activation in response to FA application is largely speculative. Despite the fact that FAs are unable to bind death receptors, they were shown to upregulate their expression in β-cells, e.g., expression of death receptor 5 (DR5, TRAILR2) [265] and TNFR (tumour necrosis factor receptor) 5 (also known as CD40) [266]. CD40 protein and/or mRNA were found upregulated, due to PA treatment in rat β-cells, human islets and islets from the HFD mice [266]. Plasma levels of its ligand (sCD40L) are elevated in people with impaired glucose tolerance, metabolic syndrome and insulin resistance [267]. CD40 upregulation due to hyperlipidaemia could thus sensitize β-cells to the death receptor pathway and caspase-8 activation in these individuals.

Nevertheless, the existence of death-ligand-independent modes of caspase-8 activation was already revealed in other cell types: e.g., ER stress-induced CHOP activation by PA application in human hepatoma cells was shown to transcriptionally upregulate DR5, which subsequently resulted in ligand-independent caspase-8 activation by this death receptor [268]. Next, under conditions of persistent ER stress, employment of the autophagosome-associated platform called iDISC/stressosome in caspase-8 activation was described recently in human colon tumour cells and in breast cancer cell lines [193]. However, whether such mechanisms may be related also to FA-induced ER stress and autophagy in β-cells is unknown. Nevertheless, the ligand-independent activation of the death receptors, e.g., DR5 receptor [268] might be also explainable by the already-demonstrated effect of saturated FAs on the activity of various membrane molecules via their effect on membrane fluidity (see Section 3).

The data available so far do not indicate an important role of the Fas receptor in lipotoxicity [269]. Surprisingly, cleavage of the Bid downstream of caspase-8 activation and the subsequent engagement of the mitochondrial pathway have not been assessed in pancreatic β-cells after FA application yet.

Aside from initiator caspase-8, initiator caspase-2 was also found to be activated by saturated FAs in β-cells, and this activation was inhibited by unsaturated FAs co-application [14,15,53,82]. Despite playing a crucial role in lipoapoptosis in some other cell types [270], silencing of caspase-2 in human β-cells did not result in a decrease of SA-induced apoptosis [82].

To conclude, experimental evidence indicates that the death receptor pathway and caspase-8 activation are also somehow involved in the mechanisms of FA-induced apoptosis and appear to be of clinical relevance in T2DM pathogenesis, as well.

### 5.3. Executioner Caspases

Executioner caspases, i.e., caspase-3, -6 and -7, are activated by proteolytic cleavage executed by initiator caspase-2, -8/10 and -9. Caspase-3 or combined caspase-3/7 activation (due to ambiguity in the detection method) was convincingly confirmed following PA and/or SA application in both human and rodent β-cells in vitro, as well as in vivo (e.g., [1,4,15,20,37,54,57,200]). HFD also led to the activation of caspase-3 in murine islets [271]. Caspase-6 and -7 activation in response to SA treatment was further shown in human β-cells [15,82,83,131]. Unsaturated FAs inhibit executioner caspases activation induced by saturated FAs in rat and human β-cell lines, in the case of their anti-apoptotic effect [15,24,82,83,131]. However, in studies in which unsaturated FAs also induce β-cell apoptosis, the activation of caspases-3 [52,57] or the activation of caspase-3 together with the activation of caspase-7 [56] was found, as well.

The question of whether the inhibition of caspases is able to prevent FA-induced apoptosis was also addressed. Experiments employing the wide spectrum caspase inhibitor z-VAD-fmk confirmed the important role of caspases in FA-induced cell death [3,11] but indicated the involvement of non-caspase proteases, as well.

The formation of specific apoptotic complexes responsible for activation of executioner caspases, such as apoptosome, PIDDosome (p53-induced protein with a death domain) and DISC, where initiator caspases-9, -2 and -8/10, respectively, are activated, has not yet been addressed during FA-induced apoptosis in pancreatic β-cells.

To summarize, executioner caspases are undoubtedly activated during FA-induced apoptosis of pancreatic β-cells and play a prominent role here. Nevertheless, non-caspase proteases are also involved to some extent.

## 6. Summarized Mechanisms of Apoptosis Induction by Fatty Acids

**Figure 1:**
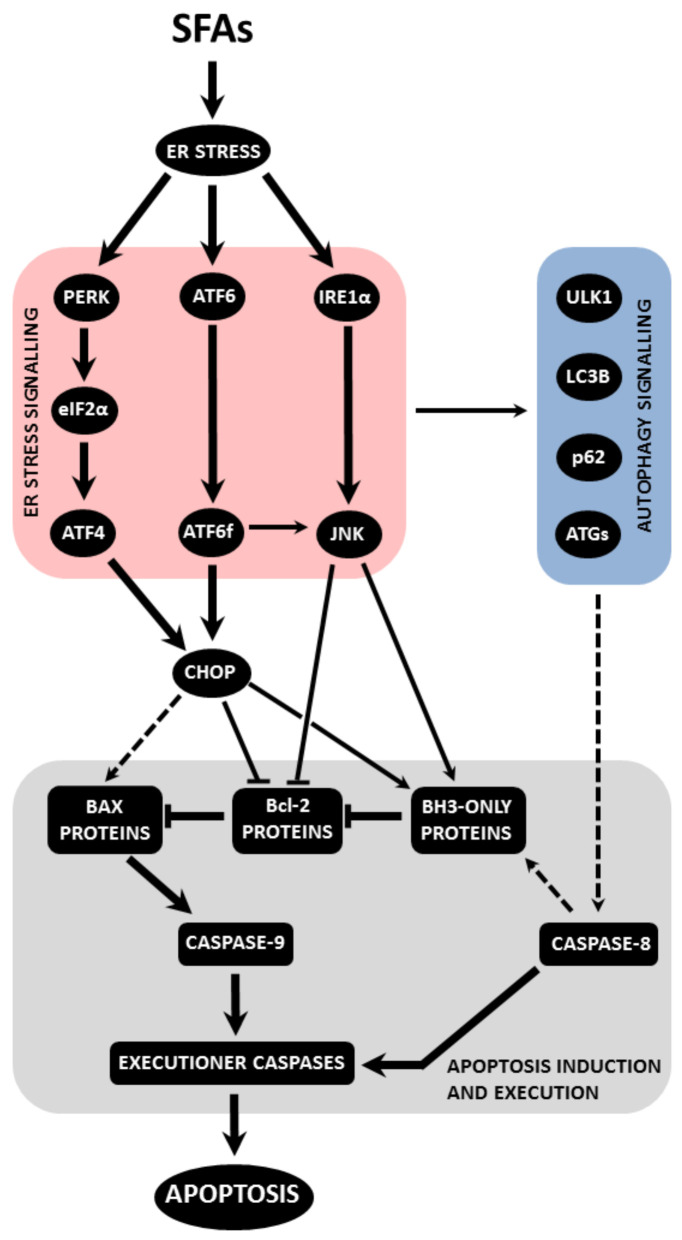
Suggested mechanisms of the possible involvement of endoplasmic reticulum (ER) stress and autophagy signalling in apoptosis induced by saturated fatty acids (SFAs) in pancreatic β-cells. Thick lines mean strong support of the respective relationship by available data, while thin lines mean weaker support. Dashed lines show a relationship proved only in other cell types.

**Figure 2:**
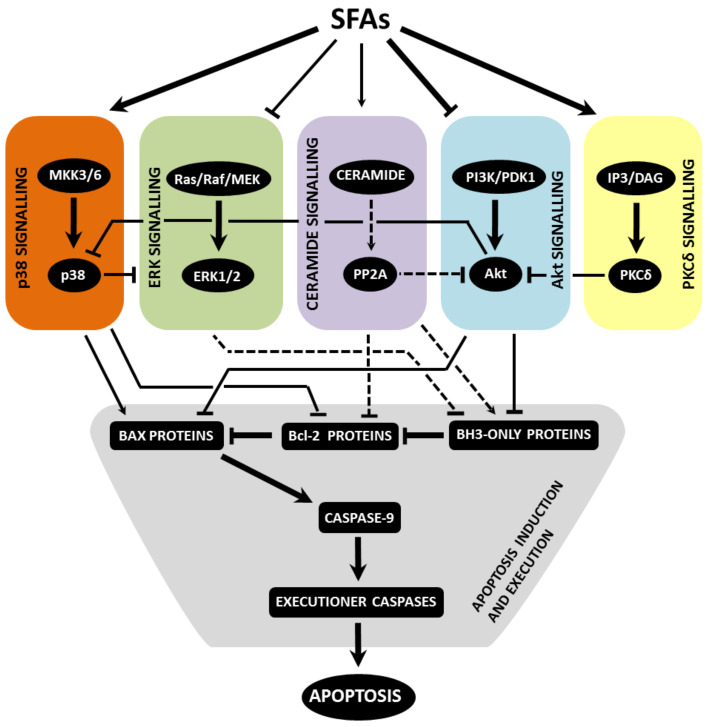
Suggested mechanisms of the possible involvement of p38 signalling, ERK signalling, ceramide signalling and Akt signalling and PKCδ signalling in apoptosis induced by saturated fatty acids (SFAs) in pancreatic β-cells. Thick lines mean strong support of the respective relationship by available data while thin lines mean weaker support. Dashed lines show a relationship proved only in other cell types.

**Figure 3:**
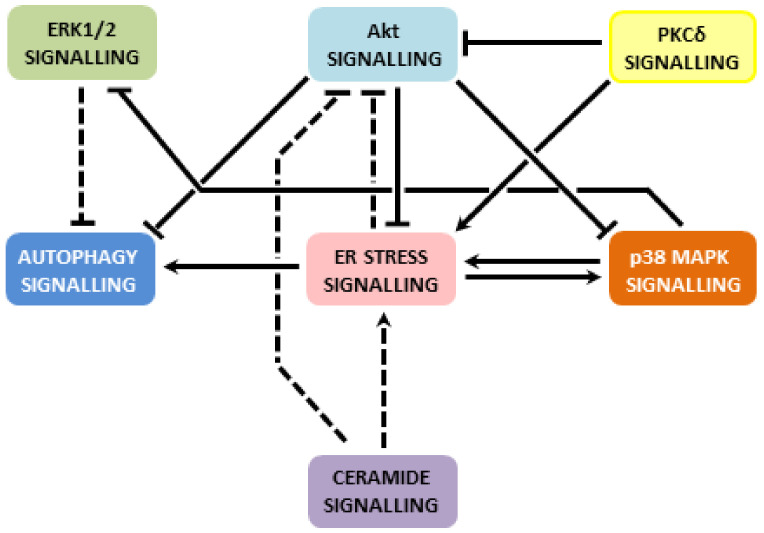
Suggested signalling pathway cross talks during apoptosis induction by saturated fatty acids in pancreatic β-cells. Dashed lines show a relationship well proved only in other cell types.

**Figure 4:**
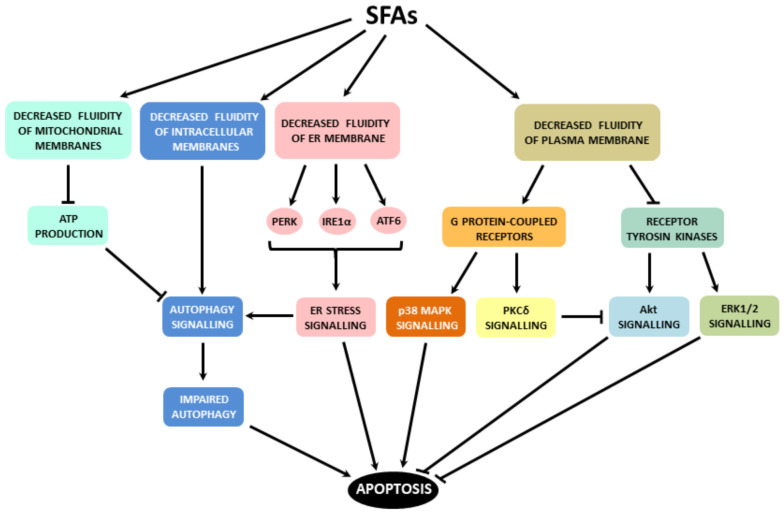
Suggested mechanisms of the possible involvement of decreased membrane fluidity in apoptosis induced by saturated fatty acids (SFAs) in pancreatic β-cells.

Saturated FAs induce ER stress in β-cells, which, in turn, leads to activation of all ER stress pathways, i.e., PERK, IRE1α and ATF6 pathway. When ER stress is severe or prolonged, apoptosis is induced. The main mediator seems to be the CHOP transcription factor. Via regulation of expression/activity of pro- and anti-apoptotic Bcl-2 family members and possibly also through the increase in ROS production, CHOP switches on the mitochondrial pathway of apoptosis induction. ER stress signalling possibly leads to autophagy signalling, which may activate caspase-8 (see also Figure 1). Saturated FAs also activate or inhibit various signalling pathways, i.e., p38 MAPK signalling, ERK signalling, ceramide signalling, Akt signalling and PKCδ signalling. This may also lead to the activation of the mitochondrial pathway of apoptosis in pancreatic β-cells (see Figure 2). Multiple cross talks within the individual above-mentioned signalling pathways were documented in β-cells or well documented in other cell types (see Figure 3). Regulation of all mentioned signalling pathways, except of ceramide signalling, might result from changed plasma or organelle membrane fluidity, due to incorporation of saturated FAs into these membranes (see Figure 4). Some miRNAs seem to play also an important role in FA-induced β-cell apoptosis.

In the rather rare situations when unsaturated FAs were also shown to be pro-apoptotic, the mechanisms mediating this effect in pancreatic β-cells seem to be the same as those described above for saturated FAs.

## 7. Mechanisms Mediating Anti-Apoptotic Effects of Unsaturated Fatty Acids

Unsaturated FAs are able to inhibit the saturated FA-induced β-cell death even when applied several hours later after the application of saturated FAs and in much lower concentrations (e.g., 50× lower) than the concentrations of saturated FAs used for cell death induction. In addition, they are effective against various other apoptotic stimuli, e.g., serum withdrawal or exposure to pro-inflammatory cytokines [6,13,24]. These findings indicate that interference at the signalling level, rather than interference with the metabolism of saturated FAs, underlies the inhibitory effect of unsaturated FAs on saturated FA-induced apoptosis [6,21,23]. This is in line with the fact that unsaturated FAs do not need to be converted to FA-CoA to exert the anti-apoptotic effect (see Section 2.3).

Concerning the direct activation of some pro-survival signalling pathway(s) by unsaturated FAs, there are very few data available. Only the Akt pathway, the classical pro-survival pathway, was suggested to be a possible mediator of the anti-apoptotic effect of unsaturated FAs against saturated FAs in rat β-cells [62]. Inhibitory phosphorylation of the forkhead transcription factor FoxO1 was proposed as a potential mediator of this effect [52]. Akt may also inhibit activation of pro-apoptotic p38 MAPK and JNK in β-cells [107] and very probably also of FoxO3 and Bad protein activation. The stimulation of Akt by unsaturated FAs may be mediated by GPR120, as documented in murine intestinal cells STC-1 for the anti-apoptotic effect of LOA against serum deprivation-induced apoptosis [272]. Moreover, GPR40, that was suggested to be involved in the protective effect of OA against PA-induced apoptosis [120], may activate Akt in β-cells [260]. However, Akt activation by unsaturated FAs in β-cells was not confirmed by others [52,120] nor by our preliminary data obtained with human β-cells NES2Y.

A lot of available data (see previous sections) indicate that the anti-apoptotic effects of unsaturated FAs may result from inhibitory intervention into pro-apoptotic signalling induced by saturated FAs. We speculate that the inhibition of the decrease of plasma and/or organelle membrane fluidity due to saturated FA incorporation may be involved here. As mentioned in Section 3, membrane fluidity decrease due to higher saturated FA incorporation can affect the activation of the membrane receptor(s) or membrane-associated proteins, which may induce pro-apoptotic signalling or inhibit pro-survival signalling. The inhibitory effect of unsaturated FAs can be generated simply by increasing membrane fluidity, which may compensate for the rigidity increase caused by saturated FAs.

Some of the possible mechanisms mediating the anti-apoptotic effect of unsaturated FAs in the cytosol might be regulated, to some extent, at the level of FA availability, as well. We speculate that saturated and unsaturated FAs may compete for membrane FA transporters, such as FABs (FAT/CD36 or fatty acid-binding proteins), which regulate cytosolic concentrations of the respective FAs.

**Figure 5:**
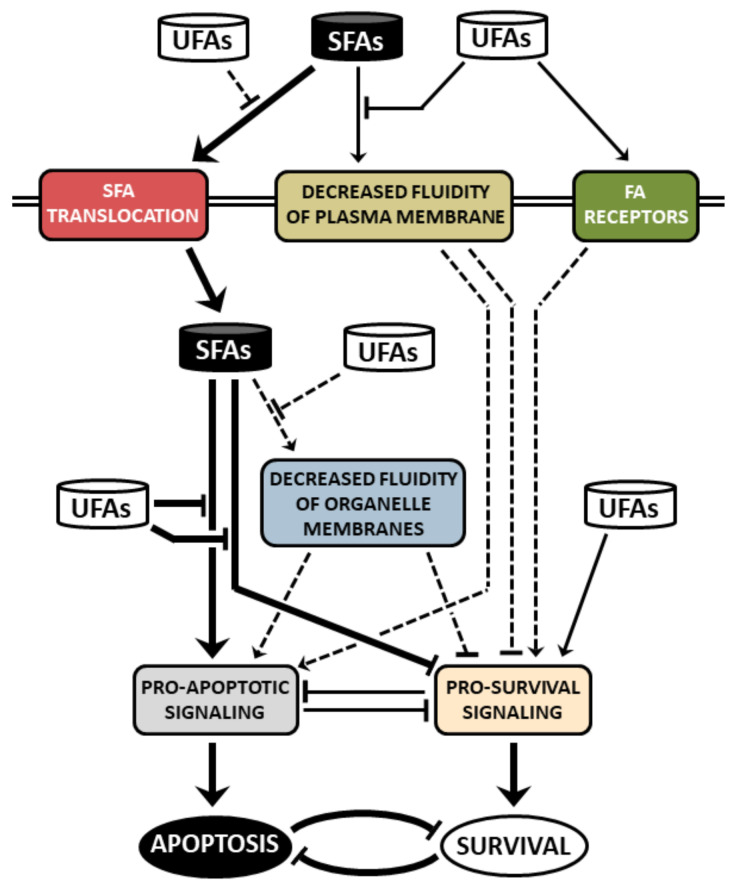
Suggested mechanisms of anti-apoptotic effects of unsaturated fatty acids (UFAs) on saturated fatty acids (SFA)-induced apoptosis in pancreatic β-cells. Thick lines mean strong support of the respective effect by available data, while thin lines mean weaker support. Dashed lines represent a hypothetical effect not proved in β-cells or in any other type of cells.

Taken together (see also Figure 5), it seems that the inhibitory effect of unsaturated FAs on saturated FA-induced apoptosis represents a inhibitory intervention into saturated FA-induced pro-apoptotic signalling rather than activation of some pro-survival signalling pathway(s) or metabolic interference in β-cells. This inhibitory intervention may result from simple counteracting the elevated membrane rigidity due to the increased saturated FA content.

## 8. Conclusions

It was clearly demonstrated that saturated FAs are able to induce apoptosis in pancreatic β-cells, whereas the pro-apoptotic effect of unsaturated FAs in β-cells is documented rather rarely. Unsaturated FAs were often shown to be even able to inhibit the pro-apoptotic effect of saturated FAs.

Despite many years of research, it is still not clear where, exactly, within the β-cell the apoptosis induction by FAs is initiated. An excess of saturated FAs and/or their chronic exposure leads to ER stress, which, if not compensated by defensive mechanisms, results in the triggering of apoptosis. The mechanisms of initiation of FA-induced apoptosis could be related to changes of membrane fluidity. Increased content of saturated FAs in cell membranes, which makes them more rigid, has already been shown to affect the activation of various plasma membrane receptors that can transmit pro-apoptotic signalling, e.g., activation of PKCδ and p38 MAPK signalling or inhibition of Akt signalling. The activity of some plasma membrane-associated proteins (e.g., c-Src) can also be affected by some FA-related processes, e.g., via protein myristoylation. Certain products of FA metabolism, e.g., ceramides, may also be involved in the initiation of pro-apoptotic signalling. As ceramides are produced preferentially from saturated FA species, this would explain at least partially the differences in the effect of saturated and unsaturated FA species.

All ER stress pathways, i.e., PERK, IRE1α and ATF6 pathways, are activated by FAs in β-cells. Nevertheless, the reason why the excess of FAs causes ER stress is rather unclear. The defensive β-cell response mainly concerns the upregulation of BiP chaperon and translation inhibition mediated by eIF2α phosphorylation. However, when ER stress is severe or prolonged, apoptosis is induced. The main mediator seems to be the CHOP transcription factor. CHOP switches on the mitochondrial pathway of apoptosis induction. ER stress pathways may also activate autophagy probably via Akt pathway inhibition.

Inhibition of the pro-survival Akt signalling seems to play an important role in the apoptosis induced by FAs. This inhibition is mediated by multiple pathways (e.g., ER stress signalling, PKCδ and ceramide) and results in, e.g., FoxO1 and FoxO3 activation, which, in turn, leads to activation of the mitochondrial pathway of apoptosis induction. Inhibition of Akt also consequences in autophagy signalling, which may then activate caspase-8. Experimental evidence also indicates the involvement of increased production of ROS, the p38 MAPK pathway and certain miRNAs (e.g., miR34a-5p and miR-297b-5p) in mechanisms of FA-induced β-cell apoptosis.

Most lines of evidence point to the activation of the mitochondrial pathway of apoptosis induction, preceded by an increased level/activity of pro-apoptotic proteins of the Bcl-2 family (Bax proteins, BH3-only proteins) and subsequent caspase-9 activation. However, the death receptor pathway of apoptosis induction and caspase-8 activation seems to play a role in FA-induced β-cell apoptosis, as well. In the rather rare situations where unsaturated FAs are also pro-apoptotic in β-cells, the mechanisms mediating this effect appear to be very similar to those described above for the saturated FAs.

Concerning the mechanism of the inhibitory effect of unsaturated FAs on saturated FA-induced β-cell apoptosis, experimental data indicate an inhibitory intervention into saturated FA-induced pro-apoptotic signalling rather than metabolic interference. Nevertheless, the point where unsaturated FAs intervene within the event/pathway leading to apoptosis induction by saturated FAs remains elusive. The activation of the Akt pathway via GPRs may be involved here. We also speculate that the inhibitory intervention may concern membrane fluidity, as unsaturated FAs exert an inverse effect here, compared to the effect of saturated FAs.

In general, understanding the mechanisms of β-cell apoptosis regulation by saturated and unsaturated FAs is of great importance, since these mechanisms seem to be shared to a great extent by various cell types. It may provide us with new possibilities of the prevention, as well as therapy of lipotoxicity-associated disorders, including diabetes.

## Abbreviations

AMPK	adenosine monophosphate-activated protein kinase
ATF6	activating transcription factor 6
Bcl-xL	B-cell lymphoma-extra large
Bcl-2	B-cell lymphoma 2
BiP	immunoglobulin heavy chain-binding protein
Bad	Bcl-2-associated death promoter
Bax	Bcl-2 associated X protein
CHOP	CCAAT-enhancer-binding protein C/EBP homologous protein
CoA	coenzyme A
DISC	death-inducing signalling complex
DP5	death protein 5
EGFR	epithelial growth factor receptor
eIF2α	eukaryotic initiation factor 2α
ER	endoplasmic reticulum
ERK	extracellular signal-regulated kinase
FA	fatty acid
FAT/CD36	fatty acid translocase/cluster of differentiation 36
FoxO	forkhead box
GPR	G protein-coupled receptor
HFD	high-fat diet
iNOS	inducible form of NOS
IRE1α	inositol-requiring protein 1α
JNK	c-Jun N-terminal kinase
LOA	linoleic acid
MAPK	mitogen-activated protein kinase
Mcl-1	myeloid cell leukemia-1
NO	nitric oxide
NOS	nitric oxide synthase
PA	palmitic acid
PERK	protein kinase RNA (PKR)-like ER kinase
PIDD	p53-induced protein with a death domain
PI3K	phosphoinositide 3-kinase
PKC	protein kinase C
POA	palmitoil acid
PUMA	p53 upregulated modulator of apoptosis
SA	stearic acid
TAG	triacylglycerol
TLR4	toll-like receptor 4
RTK	receptor tyrosine kinase
ROS	reactive oxygen species
OA	oleic acid
T2DM	type 2 diabetes mellitus
UPR	unfolded protein response
XBP1	X-box binding protein 1

## References

[1] Maedler K., Oberholzer J., Bucher P., Spinas G.A., Donath M.Y. (2003). Monounsaturated fatty acids prevent the deleterious effects of palmitate and high glucose on human pancreatic beta-cell turnover and function. Diabetes.

[2] Maedler K., Spinas G.A., Dyntar D., Moritz W., Kaiser N., Donath M.Y. (2001). Distinct effects of saturated and monounsaturated fatty acids on beta-cell turnover and function. Diabetes.

[3] Lupi R., Dotta F., Marselli L., Del Guerra S., Masini M., Santangelo C., Patane G., Boggi U., Piro S., Anello M. (2002). Prolonged exposure to free fatty acids has cytostatic and pro-apoptotic effects on human pancreatic islets: Evidence that beta-cell death is caspase mediated, partially dependent on ceramide pathway, and Bcl-2 regulated. Diabetes.

[4] El Assaad W., Buteau J., Peyot M.L., Nolan C., Roduit R., Hardy S., Joly E., Dbaibo G., Rosenberg L., Prentki M. (2003). Saturated fatty acids synergize with elevated glucose to cause pancreatic beta-cell death. Endocrinology.

[5] Kharroubi I., Ladriere L., Cardozo A.K., Dogusan Z., Cnop M., Eizirik D.L. (2004). Free fatty acids and cytokines induce pancreatic beta-cell apoptosis by different mechanisms: Role of nuclear factor-kappaB and endoplasmic reticulum stress. Endocrinology.

[6] Welters H.J., Tadayyon M., Scarpello J.H., Smith S.A., Morgan N.G. (2004). Mono-unsaturated fatty acids protect against beta-cell apoptosis induced by saturated fatty acids, serum withdrawal or cytokine exposure. FEBS Lett..

[7] Higa M., Shimabukuro M., Shimajiri Y., Takasu N., Shinjyo T., Inaba T. (2006). Protein kinase B/Akt signalling is required for palmitate-induced beta-cell lipotoxicity. Diabetes Obes. Metab..

[8] Karaskov E., Scott C., Zhang L., Teodoro T., Ravazzola M., Volchuk A. (2006). Chronic palmitate but not oleate exposure induces endoplasmic reticulum stress, which may contribute to INS-1 pancreatic beta-cell apoptosis. Endocrinology.

[9] Hennige A.M., Ranta F., Heinzelmann I., Dufer M., Michael D., Braumuller H., Lutz S.Z., Lammers R., Drews G., Bosch F. (2010). Overexpression of kinase-negative protein kinase Cdelta in pancreatic beta-cells protects mice from diet-induced glucose intolerance and beta-cell dysfunction. Diabetes.

[10] Eitel K., Staiger H., Brendel M.D., Brandhorst D., Bretzel R.G., Haring H.U., Kellerer M. (2002). Different role of saturated and unsaturated fatty acids in beta-cell apoptosis. Biochem. Biophys. Res. Commun..

[11] Welters H.J., Diakogiannaki E., Mordue J.M., Tadayyon M., Smith S.A., Morgan N.G. (2006). Differential protective effects of palmitoleic acid and cAMP on caspase activation and cell viability in pancreatic beta-cells exposed to palmitate. Apoptosis.

[12] Sone H., Kagawa Y. (2005). Pancreatic beta cell senescence contributes to the pathogenesis of type 2 diabetes in high-fat diet-induced diabetic mice. Diabetologia.

[13] Diakogiannaki E., Welters H.J., Morgan N.G. (2008). Differential regulation of the endoplasmic reticulum stress response in pancreatic beta-cells exposed to long-chain saturated and monounsaturated fatty acids. J. Endocrinol..

[14] Furstova V., Kopska T., James R.F., Kovar J. (2008). Comparison of the effect of individual saturated and unsaturated fatty acids on cell growth and death induction in the human pancreatic beta-cell line NES2Y. Life Sci..

[15] Nemcova-Furstova V., James R.F.L., Kovar J. (2011). Inhibitory Effect of Unsaturated Fatty Acids on Saturated Fatty Acid-Induced Apoptosis in Human Pancreatic beta-Cells: Activation of Caspases and ER Stress Induction. Cell. Physiol. Biochem..

[16] Oh Y.S., Bae G.D., Baek D.J., Park E.Y., Jun H.S. (2018). Fatty Acid-Induced Lipotoxicity in Pancreatic Beta-Cells During Development of Type 2 Diabetes. Front. Endocrinol..

[17] Joseph J.W., Koshkin V., Zhang C.Y., Wang J., Lowell B.B., Chan C.B., Wheeler M.B. (2002). Uncoupling protein 2 knockout mice have enhanced insulin secretory capacity after a high-fat diet. Diabetes.

[18] Sauter N.S., Schulthess F.T., Galasso R., Castellani L.W., Maedler K. (2008). The antiinflammatory cytokine interleukin-1 receptor antagonist protects from high-fat diet-induced hyperglycemia. Endocrinology.

[19] Yang L., Yao D.D., Yang H.Y., Wei Y.J., Peng Y.R., Ding Y.F., Shu L. (2016). Puerarin Protects Pancreatic beta-Cells in Obese Diabetic Mice via Activation of GLP-1R Signaling. Mol. Endocrinol..

[20] Li J., Xu S.S., Liu Y.Q., Yan Z., Zhang F., Lv Q.G., Tong N.W. (2019). Activated PPAR beta/delta Protects Pancreatic beta Cells in Type 2 Diabetic Goto-Kakizaki Rats from Lipoapoptosis via GPR40. Lipids.

[21] Morgan N.G. (2009). Fatty acids and beta-cell toxicity. Curr. Opin. Clin. Nutr. Metab. Care.

[22] Diakogiannaki E., Dhayal S., Childs C.E., Calder P.C., Welters H.J., Morgan N.G. (2007). Mechanisms involved in the cytotoxic and cytoprotective actions of saturated versus monounsaturated long-chain fatty acids in pancreatic beta-cells. J. Endocrinol..

[23] Newsholme P., Keane D., Welters H.J., Morgan N.G. (2007). Life and death decisions of the pancreatic beta-cell: The role of fatty acids. Clin. Sci..

[24] Dhayal S., Welters H.J., Morgan N.G. (2008). Structural requirements for the cytoprotective actions of mono-unsaturated fatty acids in the pancreatic beta-cell line, BRIN-BD11. Br. J. Pharm..

[25] Plotz T., von Hanstein A.S., Krummel B., Laporte A., Mehmeti I., Lenzen S. (2019). Structure-toxicity relationships of saturated and unsaturated free fatty acids for elucidating the lipotoxic effects in human EndoC-beta H1 beta-cells. Biochim. Biophys. Acta Mol. Basis Dis..

[26] Ingalls S.T., Xu Y., Hoppel C.L. (1995). Determination of plasma non-esterified fatty acids and triglyceride fatty acids by gas chromatography of their methyl esters after isolation by column chromatography on silica gel. J. Chromatogr. B Biomed. Appl..

[27] Lagerstedt S.A., Hinrichs D.R., Batt S.M., Magera M.J., Rinaldo P., McConnell J.P. (2001). Quantitative determination of plasma c8-c26 total fatty acids for the biochemical diagnosis of nutritional and metabolic disorders. Mol. Genet. Metab..

[28] Cnop M., Ladriere L., Hekerman P., Ortis F., Cardozo A.K., Dogusan Z., Flamez D., Boyce M., Yuan J., Eizirik D.L. (2007). Selective inhibition of eukaryotic translation initiation factor 2 alpha dephosphorylation potentiates fatty acid-induced endoplasmic reticulum stress and causes pancreatic beta-cell dysfunction and apoptosis. J. Biol. Chem..

[29] Liu X., Zeng X., Chen X., Luo R., Li L., Wang C., Liu J., Cheng J., Lu Y., Chen Y. (2019). Oleic acid protects insulin-secreting INS-1E cells against palmitic acid-induced lipotoxicity along with an amelioration of ER stress. Endocrine.

[30] Lai E., Bikopoulos G., Wheeler M.B., Rozakis-Adcock M., Volchuk A. (2008). Differential activation of ER stress and apoptosis in response to chronically elevated free fatty acids in pancreatic beta-cells. Am. J. Physiol. Endocrinol. Metab..

[31] Ladriere L., Igoillo-Esteve M., Cunha D.A., Brion J.P., Bugliani M., Marchetti P., Eizirik D.L., Cnop M. (2010). Enhanced signaling downstream of ribonucleic Acid-activated protein kinase-like endoplasmic reticulum kinase potentiates lipotoxic endoplasmic reticulum stress in human islets. J. Clin. Endocrinol. Metab..

[32] Thorn K., Hovsepyan M., Bergsten P. (2010). Reduced levels of SCD1 accentuate palmitate-induced stress in insulin-producing beta-cells. Lipids Health Dis..

[33] Hirota N., Otabe S., Nakayama H., Yuan X., Yamada K. (2006). Sequential activation of caspases and synergistic beta-cell cytotoxicity by palmitate and anti-Fas antibodies. Life Sci..

[34] Baldwin A.C., Green C.D., Olson L.K., Moxley M.A., Corbett J.A. (2012). A role for aberrant protein palmitoylation in FFA-induced ER stress and beta cell death. Am. J. Physiol. Endocrinol. Metab..

[35] Tuei V.C., Ha J.S., Ha C.E. (2011). Effects of human serum albumin complexed with free fatty acids on cell viability and insulin secretion in the hamster pancreatic beta-cell line HIT-T15. Life Sci..

[36] Busch A.K., Gurisik E., Cordery D.V., Sudlow M., Denyer G.S., Laybutt D.R., Hughes W.E., Biden T.J. (2005). Increased fatty acid desaturation and enhanced expression of stearoyl coenzyme A desaturase protects pancreatic beta-cells from lipoapoptosis. Diabetes.

[37] Choi S.E., Kim H.E., Shin H.C., Jang H.J., Lee K.W., Kim Y., Kang S.S., Chun J., Kang Y. (2007). Involvement of Ca2+-mediated apoptotic signals in palmitate-induced MIN6N8a beta cell death. Mol. Cell. Endocrinol..

[38] Laybutt D.R., Preston A.M., Akerfeldt M.C., Kench J.G., Busch A.K., Biankin A.V., Biden T.J. (2007). Endoplasmic reticulum stress contributes to beta cell apoptosis in type 2 diabetes. Diabetologia.

[39] Jeffrey K.D., Alejandro E.U., Luciani D.S., Kalynyak T.B., Hu X., Li H., Lin Y., Townsend R.R., Polonsky K.S., Johnson J.D. (2008). Carboxypeptidase E mediates palmitate-induced beta-cell ER stress and apoptosis. Proc. Natl. Acad. Sci. USA.

[40] Eitel K., Staiger H., Rieger J., Mischak H., Brandhorst H., Brendel M.D., Bretzel R.G., Haring H.U., Kellerer M. (2003). Protein kinase C delta activation and translocation to the nucleus are required for fatty acid-induced apoptosis of insulin-secreting cells. Diabetes.

[41] Gehrmann W., Wurdemann W., Plotz T., Jorns A., Lenzen S., Elsner M. (2015). Antagonism Between Saturated and Unsaturated Fatty Acids in ROS Mediated Lipotoxicity in Rat Insulin-Producing Cells. Cell. Physiol. Biochem..

[42] Sarnyai F., Donko M.B., Matyasi J., Gor-Nagy Z., Marczi I., Simon-Szabo L., Zambo V., Somogyi A., Csizmadia T., Low P. (2019). Cellular toxicity of dietary trans fatty acids and its correlation with ceramide and diglyceride accumulation. Food Chem. Toxicol..

[43] Okuyama R., Fujiwara T., Ohsumi J. (2003). High glucose potentiates palmitate-induced NO-mediated cytotoxicity through generation of superoxide in clonal beta-cell HIT-T15. FEBS Lett..

[44] Moffitt J.H., Fielding B.A., Evershed R., Berstan R., Currie J.M., Clark A. (2005). Adverse physicochemical properties of tripalmitin in beta cells lead to morphological changes and lipotoxicity in vitro. Diabetologia.

[45] Qi Y., Xia P. (2012). Cellular Inhibitor of Apoptosis Protein-1 (cIAP1) Plays a Critical Role in β-Cell Survival under Endoplasmic Reticulum Stress: Promoting ubiquitination and degradation of C/EBP Homologous Protein (CHOP). J. Biol. Chem..

[46] Sommerweiss D., Gorski T., Richter S., Garten A., Kiess W. (2013). Oleate rescues INS-1E beta-cells from palmitate-induced apoptosis by preventing activation of the unfolded protein response. Biochem. Biophys. Res. Commun..

[47] Vasu S., McClenaghan N.H., McCluskey J.T., Flatt P.R. (2013). Effects of lipotoxicity on a novel insulin-secreting human pancreatic beta-cell line, 1.1B4. Biol. Chem..

[48] Nemecz M., Constantin A., Dumitrescu M., Alexandru N., Filippi A., Tanko G., Georgescu A. (2018). The Distinct Effects of Palmitic and Oleic Acid on Pancreatic Beta Cell Function: The Elucidation of Associated Mechanisms and Effector Molecules. Front. Pharm..

[49] Jurado-Ruiz E., Alvarez-Amor L., Varela L.M., Berna G., Parra-Camacho M.S., Oliveras-Lopez M.J., Martinez-Force E., Rojas A., Hmadcha A., Soria B. (2019). Extra virgin olive oil diet intervention improves insulin resistance and islet performance in diet-induced diabetes in mice. Sci. Rep..

[50] Cnop M., Hannaert J.C., Hoorens A., Eizirik D.L., Pipeleers D.G. (2001). Inverse relationship between cytotoxicity of free fatty acids in pancreatic islet cells and cellular triglyceride accumulation. Diabetes.

[51] Cnop M., Hannaert J.C., Pipeleers D.G. (2002). Troglitazone does not protect rat pancreatic beta cells against free fatty acid-induced cytotoxicity. Biochem. Pharm..

[52] Wrede C.E., Dickson L.M., Lingohr M.K., Briaud I., Rhodes C.J. (2002). Protein kinase B/Akt prevents fatty acid-induced apoptosis in pancreatic beta-cells (INS-1). J. Biol. Chem..

[53] Maestre I., Jordan J., Calvo S., Reig J.A., Cena V., Soria B., Prentki M., Roche E. (2003). Mitochondrial dysfunction is involved in apoptosis induced by serum withdrawal and fatty acids in the beta-cell line INS-1. Endocrinology.

[54] Cunha D.A., Hekerman P., Ladriere L., Bazarra-Castro A., Ortis F., Wakeham M.C., Moore F., Rasschaert J., Cardozo A.K., Bellomo E. (2008). Initiation and execution of lipotoxic ER stress in pancreatic beta-cells. J. Cell Sci..

[55] Li J., Liu X., Ran X., Chen J., Li X., Wu W., Huang H., Huang H., Long Y., Liang J. (2010). Sterol regulatory element-binding protein-1c knockdown protected INS-1E cells from lipotoxicity. Diabetes Obes. Metab..

[56] Tuo Y., Wang D., Li S., Chen C. (2011). Long-term exposure of INS-1 rat insulinoma cells to linoleic acid and glucose in vitro affects cell viability and function through mitochondrial-mediated pathways. Endocrine.

[57] Plotz T., Krummel B., Laporte A., Pingitore A., Persaud S.J., Jorns A., Elsner M., Mehmeti I., Lenzen S. (2017). The monounsaturated fatty acid oleate is the major physiological toxic free fatty acid for human beta cells. Nutr. Diabetes.

[58] Dhayal S., Morgan N.G. (2011). Structure-activity relationships influencing lipid-induced changes in eIF2alpha phosphorylation and cell viability in BRIN-BD11 cells. FEBS Lett..

[59] Bellini L., Campana M., Rouch C., Chacinska M., Bugliani M., Meneyrol K., Hainault I., Lenoir V., Denom J., Veret J. (2018). Protective role of the ELOVL2/docosahexaenoic acid axis in glucolipotoxicity-induced apoptosis in rodent beta cells and human islets. Diabetologia.

[60] Sargsyan E., Artemenko K., Manukyan L., Bergquist J., Bergsten P. (2016). Oleate protects beta-cells from the toxic effect of palmitate by activating pro-survival pathways of the ER stress response. Biochim. Biophys. Acta.

[61] Plotz T., Hartmann M., Lenzen S., Elsner M. (2016). The role of lipid droplet formation in the protection of unsaturated fatty acids against palmitic acid induced lipotoxicity to rat insulin-producing cells. Nutr. Metab..

[62] Beeharry N., Chambers J.A., Green I.C. (2004). Fatty acid protection from palmitic acid-induced apoptosis is lost following PI3-kinase inhibition. Apoptosis.

[63] Zhu Y.X., Zhang X.Y., Zhang L., Zhang M.L., Li L., Luo D., Zhong Y. (2019). Perilipin5 protects against lipotoxicity and alleviates endoplasmic reticulum stress in pancreatic beta-cells. Nutr. Metab..

[64] McGarry J.D., Brown N.F. (1997). The mitochondrial carnitine palmitoyltransferase system. From concept to molecular analysis. Eur. J. Biochem..

[65] Shimabukuro M., Zhou Y.T., Levi M., Unger R.H. (1998). Fatty acid-induced beta cell apoptosis: A link between obesity and diabetes. Proc. Natl. Acad. Sci. USA.

[66] Wang X., Zhou L.B., Li G., Luo T.H., Gu Y.Y., Qian L., Fu X.L., Li F.Y., Li J.P., Luo M. (2007). Palmitate activates AMP-activated protein kinase and regulates insulin secretion from beta cells. Biochem. Biophys. Res. Commun..

[67] Fu A., Eberhard C.E., Screaton R.A. (2013). Role of AMPK in pancreatic beta cell function. Mol. Cell. Endocrinol..

[68] Dai Y.L., Huang S.L., Leng Y. (2015). AICAR and Metformin Exert AMPK-dependent Effects on INS-1E Pancreatic beta-cell Apoptosis via Differential Downstream Mechanisms. Int. J. Biol. Sci..

[69] Oshima M., Pechberty S., Bellini L., Gopel S.O., Campana M., Rouch C., Dairou J., Cosentino C., Fantuzzi F., Toivonen S. (2020). Stearoyl CoA desaturase is a gatekeeper that protects human beta cells against lipotoxicity and maintains their identity. Diabetologia.

[70] Janikiewicz J., Hanzelka K., Dziewulska A., Kozinski K., Dobrzyn P., Bernas T., Dobrzyn A. (2015). Inhibition of SCD1 impairs palmitate-derived autophagy at the step of autophagosome-lysosome fusion in pancreatic beta-cells. J. Lipid. Res..

[71] Briaud I., Harmon J.S., Kelpe C.L., Segu V.B., Poitout V. (2001). Lipotoxicity of the pancreatic beta-cell is associated with glucose-dependent esterification of fatty acids into neutral lipids. Diabetes.

[72] Listenberger L.L., Han X., Lewis S.E., Cases S., Farese R.V., Ory D.S., Schaffer J.E. (2003). Triglyceride accumulation protects against fatty acid-induced lipotoxicity. Proc. Natl. Acad. Sci. USA.

[73] Chen E., Tsai T.H., Li L., Saha P., Chan L., Chang B.H.J. (2017). PLIN2 is a Key Regulator of the Unfolded Protein Response and Endoplasmic Reticulum Stress Resolution in Pancreatic beta Cells. Sci. Rep..

[74] Kelpe C.L., Moore P.C., Parazzoli S.D., Wicksteed B., Rhodes C.J., Poitout V. (2003). Palmitate inhibition of insulin gene expression is mediated at the transcriptional level via ceramide synthesis. J. Biol. Chem..

[75] Manukyan L., Ubhayasekera S., Bergquist J., Sargsyan E., Bergsten P. (2015). Palmitate-Induced Impairments of beta-Cell Function Are Linked With Generation of Specific Ceramide Species via Acylation of Sphingosine. Endocrinology.

[76] Shimabukuro M., Higa M., Zhou Y.T., Wang M.Y., Newgard C.B., Unger R.H. (1998). Lipoapoptosis in beta-cells of obese prediabetic fa/fa rats. Role of serine palmitoyltransferase overexpression. J. Biol. Chem..

[77] Veret J., Coant N., Berdyshev E.V., Skobeleva A., Therville N., Bailbe D., Gorshkova I., Natarajan V., Portha B., Le Stunff H. (2011). Ceramide synthase 4 and de novo production of ceramides with specific N-acyl chain lengths are involved in glucolipotoxicity-induced apoptosis of INS-1 beta-cells. Biochem. J..

[78] Nikolova-Karakashian M.N., Rozenova K.A., Chalfant C., Del Poeta M. (2010). Ceramide in Stress Response. Sphingolipids as Signaling and Regulatory Molecules.

[79] Stratford S., Hoehn K.L., Liu F., Summers S.A. (2004). Regulation of insulin action by ceramide: Dual mechanisms linking ceramide accumulation to the inhibition of Akt/protein kinase B. J. Biol. Chem..

[80] Blouin C.M., Prado C., Takane K.K., Lasnier F., Garcia-Ocana A., Ferre P., Dugail I., Hajduch E. (2010). Plasma membrane subdomain compartmentalization contributes to distinct mechanisms of ceramide action on insulin signaling. Diabetes.

[81] Morales A., Lee H., Goni F.M., Kolesnick R., Fernandez-Checa J.C. (2007). Sphingolipids and cell death. Apoptosis.

[82] Nemcova-Furstova V., Balusikova K., Sramek J., James R.F., Kovar J. (2013). Caspase-2 and JNK activated by saturated fatty acids are not involved in apoptosis induction but modulate ER stress in human pancreatic beta-cells. Cell. Physiol. Biochem..

[83] Sramek J., Nemcova-Furstova V., Pavlikova N., Kovar J. (2017). Effect of Saturated Stearic Acid on MAP Kinase and ER Stress Signaling Pathways during Apoptosis Induction in Human Pancreatic beta-Cells Is Inhibited by Unsaturated Oleic Acid. Int. J. Mol. Sci..

[84] Lang F., Ullrich S., Gulbins E. (2011). Ceramide formation as a target in beta-cell survival and function. Expert Opin. Ther. Targets.

[85] Seshacharyulu P., Pandey P., Datta K., Batra S.K. (2013). Phosphatase: PP2A structural importance, regulation and its aberrant expression in cancer. Cancer Lett..

[86] Thevissen K., Francois I.E., Winderickx J., Pannecouque C., Cammue B.P. (2006). Ceramide involvement in apoptosis and apoptotic diseases. Mini. Rev. Med. Chem..

[87] Karnovsky M.J., Kleinfeld A.M., Hoover R.L., Klausner R.D. (1982). The concept of lipid domains in membranes. J. Cell Biol..

[88] Maulucci G., Cohen O., Daniel B., Sansone A., Petropoulou P.I., Filou S., Spyridonidis A., Pani G., De Spirito M., Chatgilialoglu C. (2016). Fatty acid-related modulations of membrane fluidity in cells: Detection and implications. Free Radic. Res..

[89] Yang X., Sheng W., Sun G.Y., Lee J.C. (2011). Effects of fatty acid unsaturation numbers on membrane fluidity and alpha-secretase-dependent amyloid precursor protein processing. Neurochem. Int..

[90] Pilon M. (2016). Revisiting the membrane-centric view of diabetes. Lipids Health Dis..

[91] Mamedova L.K., Yuan K., Laudick A.N., Fleming S.D., Mashek D.G., Bradford B.J. (2013). Toll-like receptor 4 signaling is required for induction of gluconeogenic gene expression by palmitate in human hepatic carcinoma cells. J. Nutr. Biochem..

[92] Fang Q.L., Zou C.P., Zhong P., Lin F., Li W.X., Wang L.T., Zhang Y.L., Zheng C., Wang Y., Li X.K. (2016). EGFR mediates hyperlipidemia-induced renal injury via regulating inflammation and oxidative stress: The detrimental role and mechanism of EGFR activation. Oncotarget.

[93] Vacaresse N., Lajoie-Mazenc I., Auge N., Suc I., Frisach M.F., Salvayre R., Negre-Salvayre A. (1999). Activation of epithelial growth factor receptor pathway by unsaturated fatty acids. Circ. Res..

[94] Chen B.E., Sun Y., Niu J.X., Jarugumilli G.K., Wu X. (2018). Protein Lipidation in Cell Signaling and Diseases: Function, Regulation, and Therapeutic Opportunities. Cell Chem. Biol..

[95] Holzer R.G., Park E.J., Li N., Tran H., Chen M., Choi C., Solinas G., Karin M. (2011). Saturated fatty acids induce c-Src clustering within membrane subdomains, leading to JNK activation. Cell.

[96] Manning B.D., Cantley L.C. (2007). AKT/PKB signaling: Navigating downstream. Cell.

[97] Li X.J., Guo Q.H., Wang X., Xue B., Sun L.Q., Meng Q.T., Lu J.M., Mu Y.M. (2012). LRP16 gene protects mouse insulinoma MIN6 cells against fatty acid-induced apoptosis through Akt/FoxO1 signaling. Chin. Med. J..

[98] Quan X., Zhang L., Li Y., Liang C. (2014). TCF2 attenuates FFA-induced damage in islet beta-cells by regulating production of insulin and ROS. Int. J. Mol. Sci..

[99] Shao S., Nie M., Chen C., Chen X., Zhang M., Yuan G., Yu X., Yang Y. (2014). Protective action of liraglutide in beta cells under lipotoxic stress via PI3K/Akt/FoxO1 pathway. J. Cell. Biochem..

[100] Hao F., Kang J., Cao Y., Fan S., Yang H., An Y., Pan Y., Tie L., Li X. (2015). Curcumin attenuates palmitate-induced apoptosis in MIN6 pancreatic beta-cells through PI3K/Akt/FoxO1 and mitochondrial survival pathways. Apoptosis.

[101] Natalicchio A., Marrano N., Biondi G., Spagnuolo R., Labarbuta R., Porreca I., Cignarelli A., Bugliani M., Marchetti P., Perrini S. (2017). The Myokine Irisin Is Released in Response to Saturated Fatty Acids and Promotes Pancreatic beta-Cell Survival and Insulin Secretion. Diabetes.

[102] Litwak S.A., Wali J.A., Pappas E.G., Saadi H., Stanley W.J., Varanasi L.C., Kay T.W.H., Thomas H.E., Gurzov E.N. (2015). Lipotoxic Stress Induces Pancreatic beta-Cell Apoptosis through Modulation of Bcl-2 Proteins by the Ubiquitin-Proteasome System. J. Diabetes Res..

[103] Cunha D.A., Gurzov E.N., Naamane N., Ortis F., Cardozo A.K., Bugliani M., Marchetti P., Eizirik D.L., Cnop M. (2014). JunB protects beta-cells from lipotoxicity via the XBP1-AKT pathway. Cell Death Differ..

[104] Buteau J., El-Assaad W., Rhodes C.J., Rosenberg L., Joly E., Prentki M. (2004). Glucagon-like peptide-1 prevents beta cell glucolipotoxicity. Diabetologia.

[105] Tuttle R.L., Gill N.S., Pugh W., Lee J.P., Koeberlein B., Furth E.E., Polonsky K.S., Naji A., Birnbaum M.J. (2001). Regulation of pancreatic beta-cell growth and survival by the serine/threonine protein kinase Akt1/PKB alpha. Nat. Med..

[106] Bernal-Mizrachi E., Fatrai S., Johnson J.D., Ohsugi M., Otani K., Han Z.Q., Polonsky K.S., Permutt M.A. (2004). Defective insulin secretion and increased susceptibility to experimental diabetes are induced by reduced Akt activity in pancreatic islet beta cells. J. Clin. Investig..

[107] Widenmaier S.B., Ao Z.L., Kim S.J., Warnock G., McIntosh C.H.S. (2009). Suppression of p38 MAPK and JNK via Akt-mediated Inhibition of Apoptosis Signal-regulating Kinase 1 Constitutes a Core Component of the beta-Cell Pro-survival Effects of Glucose-dependent Insulinotropic Polypeptide. J. Biol. Chem..

[108] Aikin R., Maysinger D., Rosenberg L. (2004). Cross-talk between phosphatidylinositol 3-kinase/AKT and c-Jun NH2-terminal kinase mediates survival of isolated human islets. Endocrinology.

[109] Zhang X.B., Tang N.M., Hadden T.J., Rishi A.K. (2011). Akt, FoxO and regulation of apoptosis. Biochim. Biophys. Acta Mol. Cell Res..

[110] Kitamura T., Kitamura Y.I. (2007). Role of FoxO proteins in pancreatic beta cells. Endocr. J..

[111] Kitamura T., Nakae J., Kitamura Y., Kido Y., Biggs W.H., Wright C.V.E., White M.F., Arden K.C., Accili D. (2002). The forkhead transcription factor Foxo1 links insulin signaling to Pdx1 regulation of pancreatic beta cell growth. J. Clin. Investig..

[112] Huang H., Tindall J.T. (2006). FOXO factors: A matter of life and death. Future Oncol..

[113] Wang W., Liu Y., Chen Y., Cao C., Xiang Y., Zhang D., Han L., Zhao H., Liu G. (2010). Inhibition of Foxo1 mediates protective effects of ghrelin against lipotoxicity in MIN6 pancreatic beta-cells. Peptides.

[114] Yuan H., Zhang X., Huang X., Lu Y., Tang W., Man Y., Wang S., Xi J., Li J. (2010). NADPH oxidase 2-derived reactive oxygen species mediate FFAs-induced dysfunction and apoptosis of beta-cells via JNK, p38 MAPK and p53 pathways. PLoS ONE.

[115] Yin Y., Yong W., Yu J., Zhang X., Lin H., Zhu Y., Han X. (2016). Pdcd2l Promotes Palmitate-Induced Pancreatic Beta-Cell Apoptosis as a FoxO1 Target Gene. PLoS ONE.

[116] Kim S.J., Winter K., Nian C., Tsuneoka M., Koda Y., McIntosh C.H. (2005). Glucose-dependent insulinotropic polypeptide (GIP) stimulation of pancreatic beta-cell survival is dependent upon phosphatidylinositol 3-kinase (PI3K)/protein kinase B (PKB) signaling, inactivation of the forkhead transcription factor Foxo1, and down-regulation of bax expression. J. Biol. Chem..

[117] Martinez S.C., Tanabe K., Cras-Meneur C., Abumrad N.A., Bernal-Mizrachi E., Permutt M.A. (2008). Inhibition of Foxo1 protects pancreatic islet beta-cells against fatty acid and endoplasmic reticulum stress-induced apoptosis. Diabetes.

[118] Cunha D.A., Igoillo-Esteve M., Gurzov E.N., Germano C.M., Naamane N., Marhfour I., Fukaya M., Vanderwinden J.M., Gysemans C., Mathieu C. (2012). Death Protein 5 and p53-Upregulated Modulator of Apoptosis Mediate the Endoplasmic Reticulum Stress-Mitochondrial Dialog Triggering Lipotoxic Rodent and Human beta-Cell Apoptosis. Diabetes.

[119] Wrede C.E., Dickson L.M., Lingohr M.K., Briaud I., Rhodes C.J. (2003). Fatty acid and phorbol ester-mediated interference of mitogenic signaling via novel protein kinase C isoforms in pancreatic beta-cells (INS-1). J. Mol. Endocrinol..

[120] Zhang Y., Xu M., Zhang S., Yan L., Yang C., Lu W., Li Y., Cheng H. (2007). The role of G protein-coupled receptor 40 in lipoapoptosis in mouse beta-cell line NIT-1. J. Mol. Endocrinol..

[121] Simon M.N., Azevedo-Martins A.K., Amanso A.M., Carvalho C.R.O., Curi R. (2008). Persistent activation of Akt or ERK prevents the toxicity induced by saturated and polyunsaturated fatty acids in RINm5F beta-cells. Toxicol. Vitr..

[122] Chang F., Steelman L.S., Lee J.T., Shelton J.G., Navolanic P.M., Blalock W.L., Franklin R.A., McCubrey J.A. (2003). Signal transduction mediated by the Ras/Raf/MEK/ERK pathway from cytokine receptors to transcription factors: Potential targeting for therapeutic intervention. Leukemia.

[123] Roskoski R. (2012). ERK1/2 MAP kinases: Structure, function, and regulation. Pharm. Res..

[124] Panse M., Gerst F., Kaiser G., Teutsch C.A., Dolker R., Wagner R., Haring H.U., Ullrich S. (2015). Activation of extracellular signal-regulated protein kinases 1 and 2 (ERK1/2) by free fatty acid receptor 1 (FFAR1/GPR40) protects from palmitate-induced beta cell death, but plays no role in insulin secretion. Cell. Physiol. Biochem..

[125] Abaraviciene S.M., Lundquist I., Salehi A. (2008). Rosiglitazone counteracts palmitate-induced beta-cell dysfunction by suppression of MAP kinase, inducible nitric oxide synthase and caspase 3 activities. Cell. Mol. Life Sci..

[126] Fontes G., Semache M., Hagman D.K., Tremblay C., Shah R., Rhodes C.J., Rutter J., Poitout V. (2009). Involvement of Per-Arnt-Sim Kinase and Extracellular-Regulated Kinases-1/2 in Palmitate Inhibition of Insulin Gene Expression in Pancreatic beta-Cells. Diabetes.

[127] Plaisance V., Perret V., Favre D., Abderrahmani A., Yang J.Y., Widmann C., Regazzi R. (2009). Role of the transcriptional factor C/EBPbeta in free fatty acid-elicited beta-cell failure. Mol. Cell. Endocrinol..

[128] Guo J., Qian Y., Xi X., Hu X., Zhu J., Han X. (2010). Blockage of ceramide metabolism exacerbates palmitate inhibition of pro-insulin gene expression in pancreatic beta-cells. Mol. Cell. Biochem..

[129] Watson M.L., Macrae K., Marley A.E., Hundal H.S. (2011). Chronic effects of palmitate overload on nutrient-induced insulin secretion and autocrine signalling in pancreatic MIN6 beta cells. PLoS ONE.

[130] Liu L., Liang C., Mei P.C., Zhu H., Hou M.L., Yu C.L., Song Z.B., Bao Y.L., Huang Y.X., Yi J.W. (2019). Dracorhodin perchlorate protects pancreatic beta-cells against glucotoxicity- or lipotoxicity-induced dysfunction and apoptosis in vitro and in vivo. FEBS J..

[131] Sramek J., Nemcova-Furstova V., Kovar J. (2016). Kinase Signaling in Apoptosis Induced by Saturated Fatty Acids in Pancreatic beta-Cells. Int. J. Mol. Sci..

[132] Morgan N.G., Dhayal S., Diakogiannaki E., Welters H.J. (2008). The cytoprotective actions of long-chain mono-unsaturated fatty acids in pancreatic beta-cells. Biochem. Soc. Trans..

[133] Cagnol S., Chambard J.C. (2010). ERK and cell death: Mechanisms of ERK-induced cell death—Apoptosis, autophagy and senescence. FEBS J..

[134] Lu Z., Xu S. (2006). ERK1/2 MAP kinases in cell survival and apoptosis. IUBMB Life.

[135] Lavallard V.J., Meijer A.J., Codogno P., Gual P. (2012). Autophagy, signaling and obesity. Pharm. Res..

[136] Zarubin T., Han J.H. (2005). Activation and signaling of the p38 MAP kinase pathway. Cell Res..

[137] Cvjeticanin T., Stojanovic I., Timotijevic G., Stosic-Grujicic S., Miljkovic D. (2009). T cells cooperate with palmitic acid in induction of beta cell apoptosis. BMC Immunol..

[138] Natalicchio A., Labarbuta R., Tortosa F., Biondi G., Marrano N., Peschechera A., Carchia E., Orlando M.R., Leonardini A., Cignarelli A. (2013). Exendin-4 protects pancreatic beta cells from palmitate-induced apoptosis by interfering with GPR40 and the MKK4/7 stress kinase signalling pathway. Diabetologia.

[139] Wang Y., Xie T., Zhang D., Leung P.S. (2019). GPR120 protects lipotoxicity- induced pancreatic beta-cell dysfunction through regulation of PDX1 expression and inhibition of islet inflammation. Clin. Sci..

[140] Zhou L., Cai X., Han X., Ji L. (2014). P38 plays an important role in glucolipotoxicity-induced apoptosis in INS-1 cells. J. Diabetes Res..

[141] Wei X., Gu N., Feng N., Guo X., Ma X. (2018). Inhibition of p38 mitogen-activated protein kinase exerts a hypoglycemic effect by improving beta cell function via inhibition of beta cell apoptosis in db/db mice. J. Enzym. Inhib. Med. Chem..

[142] Flores-Lopez L.A., Diaz-Flores M., Garcia-Macedo R., Avalos-Rodriguez A., Vergara-Onofre M., Cruz M., Contreras-Ramos A., Konigsberg M., Ortega-Camarillo C. (2013). High glucose induces mitochondrial p53 phosphorylation by p38 MAPK in pancreatic RINm5F cells. Mol. Biol. Rep..

[143] Kikkawa U., Matsuzaki H., Yamamoto T. (2002). Protein kinase C delta (PKC delta): Activation mechanisms and functions. J. Biochem..

[144] Qin J., Fang N., Lou J.N., Zhang W.J., Xu S.Q., Liu H.L., Fang Q., Wang Z., Liu J., Men X.L. (2014). TRB3 Is Involved in Free Fatty Acid-Induced INS-1-Derived Cell Apoptosis via the Protein Kinase C delta Pathway. PLoS ONE.

[145] Welters H.J., Smith S.A., Tadayyon M., Scarpello J.H.B., Morgan N.G. (2004). Evidence that protein kinase C delta is not required for palmitate-induced cytotoxicity in BRIN-BD11 beta-cells. J. Mol. Endocrinol..

[146] Reyland M.E. (2007). Protein kinase C delta and apoptosis. Biochem. Soc. Trans..

[147] Hetz C., Martinon F., Rodriguez D., Glimcher L.H. (2011). The unfolded protein response: Integrating stress signals through the stress sensor IRE1alpha. Physiol. Rev..

[148] Tabas I., Ron D. (2011). Integrating the mechanisms of apoptosis induced by endoplasmic reticulum stress. Nat. Cell Biol..

[149] De la Cadena S.G., Massieu L. (2016). Caspases and their role in inflammation and ischemic neuronal death. Focus on caspase-12. Apoptosis.

[150] Huang C.J., Lin C.Y., Haataja L., Gurlo T., Butler A.E., Rizza R.A., Butler P.C. (2007). High expression rates of human islet amyloid polypeptide induce endoplasmic reticulum stress-mediated beta-cell apoptosis, a characteristic of humans with type 2 but not type 1 diabetes. Diabetes.

[151] Sharma R.B., Snyder J.T., Alonso L.C. (2019). Atf6 alpha impacts cell number by influencing survival, death and proliferation. Mol. Metab..

[152] Martino L., Masini M., Novelli M., Beffy P., Bugliani M., Marselli L., Masiello P., Marchetti P., De Tata V. (2012). Palmitate activates autophagy in INS-1E beta-cells and in isolated rat and human pancreatic islets. PLoS ONE.

[153] Acosta-Montano P., Rodriguez-Velazquez E., Ibarra-Lopez E., Frayde-Gomez H., Mas-Oliva J., Delgado-Coello B., Rivero I.A., Alatorre-Meda M., Aguilera J., Guevara-Olaya L. (2019). Fatty Acid and Lipopolysaccharide Effect on Beta Cells Proteostasis and its Impact on Insulin Secretion. Cells.

[154] Cunha D.A., Ladriere L., Ortis F., Igoillo-Esteve M., Gurzov E.N., Lupi R., Marchetti P., Eizirik D.L., Cnop M. (2009). Glucagon-like peptide-1 agonists protect pancreatic beta-cells from lipotoxic endoplasmic reticulum stress through upregulation of BiP and JunB. Diabetes.

[155] Bachar E., Ariav Y., Ketzinel-Gilad M., Cerasi E., Kaiser N., Leibowitz G. (2009). Glucose amplifies fatty acid-induced endoplasmic reticulum stress in pancreatic beta-cells via activation of mTORC1. PLoS ONE.

[156] Chen J.Q., Fontes G., Saxena G., Poitout V., Shalev A. (2010). Lack of TXNIP Protects Against Mitochondria-Mediated Apoptosis but Not Against Fatty Acid-Induced ER Stress-Mediated beta-Cell Death. Diabetes.

[157] Gwiazda K.S., Yang T.L., Lin Y., Johnson J.D. (2009). Effects of palmitate on ER and cytosolic Ca2+ homeostasis in beta-cells. Am. J. Physiol. Endocrinol. Metab..

[158] Sargsyan E., Sol E.R.M., Bergsten P. (2011). UPR in palmitate-treated pancreatic beta-cells is not affected by altering oxidation of the fatty acid. Nutr. Metab..

[159] Abdulkarim B., Hernangomez M., Igoillo-Esteve M., Cunha D.A., Marselli L., Marchetti P., Ladriere L., Cnop M. (2017). Guanabenz Sensitizes Pancreatic beta Cells to Lipotoxic Endoplasmic Reticulum Stress and Apoptosis. Endocrinology.

[160] Jiang D., Wan F. (2018). Exendin-4 protects INS-1 cells against palmitate-induced apoptosis through the IRE1alpha-Xbp1 signaling pathway. Exp. Med..

[161] Allagnat F., Cunha D., Moore F., Vanderwinden J.M., Eizirik D.L., Cardozo A.K. (2011). Mcl-1 downregulation by pro-inflammatory cytokines and palmitate is an early event contributing to beta-cell apoptosis. Cell Death. Differ..

[162] Lu H.M., Hao L.Y., Li S.T., Lin S., Lv L., Chen Y., Cui H.L., Zi T.Q., Chu X., Na L.X. (2016). Elevated circulating stearic acid leads to a major lipotoxic effect on mouse pancreatic beta cells in hyperlipidaemia via a miR-34a-5p-mediated PERK/p53-dependent pathway. Diabetologia.

[163] Kong F.J., Wu J.H., Sun S.Y., Zhou J.Q. (2017). The endoplasmic reticulum stress/autophagy pathway is involved in cholesterol-induced pancreatic beta-cell injury. Sci. Rep..

[164] Bugliani M., Mossuto S., Grano F., Suleiman M., Marselli L., Boggi U., De Simone P., Eizirik D.L., Cnop M., Marchetti P. (2019). Modulation of Autophagy Influences the Function and Survival of Human Pancreatic Beta Cells Under Endoplasmic Reticulum Stress Conditions and in Type 2 Diabetes. Front. Endocrinol..

[165] Ishigaki S., Fonseca S.G., Oslowski C.M., Jurczyk A., Shearstone J.R., Zhu L.J., Permutt M.A., Greiner D.L., Bortell R., Urano F. (2010). AATF mediates an antiapoptotic effect of the unfolded protein response through transcriptional regulation of AKT1. Cell Death Differ..

[166] Allagnat F., Christulia F., Ortis F., Pirot P., Lortz S., Lenzen S., Eizirik D.L., Cardozo A.K. (2010). Sustained production of spliced X-box binding protein 1 (XBP1) induces pancreatic beta cell dysfunction and apoptosis. Diabetologia.

[167] Miani M., Colli M.L., Ladriere L., Cnop M., Eizirik D.L. (2012). Mild endoplasmic reticulum st XBP1) induces pancreatic beta cell dysfunction ress augments the proinflammatory effect of IL-1beta in pancreatic rat beta-cells via the IRE1alpha/XBP1s pathway. Endocrinology.

[168] Biden T.J., Boslem E., Chu K.Y., Sue N. (2014). Lipotoxic endoplasmic reticulum stress, beta cell failure, and type 2 diabetes mellitus. Trends Endocrinol. Metab..

[169] Chan J.Y., Luzuriaga J., Maxwell E.L., West P.K., Bensellam M., Laybutt D.R. (2015). The balance between adaptive and apoptotic unfolded protein responses regulates beta-cell death under ER stress conditions through XBP1, CHOP and JNK. Mol. Cell. Endocrinol..

[170] Kim I.R., Murakami K., Chen N.J., Saibil S.D., Matysiak-Zablocki E., Elford A.R., Bonnard M., Benchimol S., Jurisicova A., Yeh W.C. (2009). DNA damage- and stress-induced apoptosis occurs independently of PIDD. Apoptosis.

[171] Oh Y.S., Lee Y.J., Kang Y., Han J., Lim O.K., Jun H.S. (2013). Exendin-4 inhibits glucolipotoxic ER stress in pancreatic beta cells via regulation of SREBP1c and C/EBP beta transcription factors. J. Endocrinol..

[172] Lee J.H., Jung I.R., Choi S.E., Lee S.M., Lee S.J., Han S.J., Kim H.J., Kim D.J., Lee K.W., Karig Y. (2014). Toxicity generated through inhibition of pyruvate carboxylase and carnitine palmitoyl transferase-1 is similar to high glucose/palmitate-induced glucolipotoxicity in INS-1 beta cells. Mol. Cell. Endocrinol..

[173] Kitahara A., Takahashi K., Morita N., Murashima T., Onuma H., Sumitani Y., Tanaka T., Kondo T., Hosaka T., Ishida H. (2017). The Novel Mechanisms Concerning the Inhibitions of Palmitate-Induced Proinflammatory Factor Releases and Endogenous Cellular Stress with Astaxanthin on MIN6-Cells. Mar. Drugs.

[174] Prause M., Christensen D.P., Billestrup N., Mandrup-Poulsen T. (2014). JNK1 Protects against Glucolipotoxicity-Mediated Beta-Cell Apoptosis. PLoS ONE.

[175] Komiya K., Uchida T., Ueno T., Koike M., Abe H., Hirose T., Kawamori R., Uchiyama Y., Kominami E., Fujitani Y. (2010). Free fatty acids stimulate autophagy in pancreatic beta-cells via JNK pathway. Biochem. Biophys. Res. Commun..

[176] Lanuza-Masdeu J., Arevalo M.I., Vila C., Barbera A., Gomis R., Caelles C. (2013). In vivo JNK activation in pancreatic beta-cells leads to glucose intolerance caused by insulin resistance in pancreas. Diabetes.

[177] Kim W.H., Lee J.W., Gao B., Jung M.H. (2005). Synergistic activation of JNK/SAPK induced by TNF-alpha and IFN-gamma: Apoptosis of pancreatic beta-cells via the p53 and ROS pathway. Cell. Signal..

[178] Lee S.M., Choi S.E., Lee J.H., Lee J.J., Jung I.R., Lee S.J., Lee K.W., Kang Y. (2011). Involvement of the TLR4 (Toll-like receptor4) signaling pathway in palmitate-induced INS-1 beta cell death. Mol. Cell. Biochem..

[179] Sramek J., Nemcova-Furstova V., Polak J., Kovar J. (2019). Hypoxia Modulates Effects of Fatty Acids on NES2Y Human Pancreatic beta-cells. Int. J. Mol. Sci..

[180] Cui W., Ma J., Wang X., Yang W., Zhang J., Ji Q. (2013). Free fatty acid induces endoplasmic reticulum stress and apoptosis of beta-cells by Ca2+/calpain-2 pathways. PLoS ONE.

[181] Teodoro T., Odisho T., Sidorova E., Volchuk A. (2012). Pancreatic beta-cells depend on basal expression of active ATF6alpha-p50 for cell survival even under nonstress conditions. Am. J. Physiol. Cell Physiol..

[182] Engin F., Yermalovich A., Nguyen T., Hummasti S., Fu W., Eizirik D.L., Mathis D., Hotamisligil G.S. (2013). Restoration of the unfolded protein response in pancreatic beta cells protects mice against type 1 diabetes. Sci. Transl. Med..

[183] Ahowesso C., Black P.N., Saini N., Montefusco D., Chekal J., Malosh C., Lindsley C.W., Stauffer S.R., DiRusso C.C. (2015). Chemical inhibition of fatty acid absorption and cellular uptake limits lipotoxic cell death. Biochem. Pharm..

[184] Ning B., Bai M.J., Shen W. (2011). Reduced Glutathione Protects Human Hepatocytes from Palmitate-Mediated Injury by Suppressing Endoplasmic Reticulum Stress Response. Hepato Gastroenterol..

[185] Kim K., Chung M.H., Park S., Cha J., Baek J.H., Lee S.Y., Choi S.Y. (2018). ER stress attenuation by Aloe-derived polysaccharides in the protection of pancreatic beta-cells from free fatty acid-induced lipotoxicity. Biochem. Biophys. Res. Commun..

[186] Song B., Scheuner D., Ron D., Pennathur S., Kaufman R.J. (2008). Chop deletion reduces oxidative stress, improves beta cell function, and promotes cell survival in multiple mouse models of diabetes. J. Clin. Investig..

[187] Eizirik D.L., Cardozo A.K., Cnop M. (2008). The role for endoplasmic reticulum stress in diabetes mellitus. Endocr. Rev..

[188] McCullough K.D., Martindale J.L., Klotz L.O., Aw T.Y., Holbrook N.J. (2001). Gadd153 sensitizes cells to endoplasmic reticulum stress by down-regulating Bcl2 and perturbing the cellular redox state. Mol. Cell. Biol..

[189] Li Y., Guo Y., Tang J., Jiang J., Chen Z. (2014). New insights into the roles of CHOP-induced apoptosis in ER stress. Acta Biochim. Biophys. Sin..

[190] Mihailidou C., Papavassiliou A.G., Kiaris H. (2014). A crosstalk between p21 and UPR-induced transcription factor C/EBP homologous protein (CHOP) linked to type 2 diabetes. Biochimie.

[191] Wali J.A., Rondas D., McKenzie M.D., Zhao Y., Elkerbout L., Fynch S., Gurzov E.N., Akira S., Mathieu C., Kay T.W.H. (2014). The proapoptotic BH3-only proteins Bim and Puma are downstream of endoplasmic reticulum and mitochondrial oxidative stress in pancreatic islets in response to glucotoxicity. Cell Death Dis..

[192] Periyasamy-Thandavan S., Jiang M., Schoenlein P., Dong Z. (2009). Autophagy: Molecular machinery, regulation, and implications for renal pathophysiology. Am. J. Physiol. Ren. Physiol..

[193] Iurlaro R., Munoz-Pinedo C. (2016). Cell death induced by endoplasmic reticulum stress. FEBS J..

[194] Yin J.J., Li Y.B., Wang Y., Liu G.D., Wang J., Zhu X.O., Pan S.H. (2012). The role of autophagy in endoplasmic reticulum stress-induced pancreatic beta cell death. Autophagy.

[195] Magnuson B., Ekim B., Fingar D.C. (2012). Regulation and function of ribosomal protein S6 kinase (S6K) within mTOR signalling networks. Biochem. J..

[196] Qi Z.H., Chen L.X., Qin Z.H. (2019). Endoplasmic Reticulum Stress and Autophagy. Autophagy: Biology and Diseases: Basic Science.

[197] Yuan T., Lupse B., Maedler K., Ardestani A. (2018). mTORC2 Signaling: A Path for Pancreatic beta Cell’s Growth and Function. J. Mol. Biol..

[198] Song S.L., Tan J., Miao Y.Y., Zhang Q. (2018). Crosstalk of ER stress-mediated autophagy and ER-phagy: Involvement of UPR and the core autophagy machinery. J. Cell. Physiol..

[199] Hatanaka M., Maier B., Sims E.K., Templin A.T., Kulkarni R.N., Evans-Molina C., Mirmira R.G. (2014). Palmitate Induces mRNA Translation and Increases ER Protein Load in Islet beta-Cells via Activation of the Mammalian Target of Rapamycin Pathway. Diabetes.

[200] Mir S.U., George N.M., Zahoor L., Harms R., Guinn Z., Sarvetnick N.E. (2015). Inhibition of autophagic turnover in beta-cells by fatty acids and glucose leads to apoptotic cell death. J. Biol. Chem..

[201] Choi S.E., Lee S.M., Lee Y.J., Li L.J., Lee S.J., Lee J.H., Kim Y., Jun H.S., Lee K.W., Kang Y. (2009). Protective role of autophagy in palmitate-induced INS-1 beta-cell death. Endocrinology.

[202] Ebato C., Uchida T., Arakawa M., Komatsu M., Ueno T., Komiya K., Azuma K., Hirose T., Tanaka K., Kominami E. (2008). Autophagy Is Important in Islet Homeostasis and Compensatory Increase of Beta Cell Mass in Response to High-Fat Diet. Cell Metab..

[203] Jung H.S., Chung K.W., Kim J.W., Kim J., Komatsu M., Tanaka K., Nguyen Y.H., Kang T.M., Yoon K.H., Kim J.W. (2008). Loss of Autophagy Diminishes Pancreatic beta Cell Mass and Function with Resultant Hyperglycemia. Cell Metab..

[204] Kaniuk N.A., Kiraly M., Bates H., Vranic M., Volchuk A., Brumell J.H. (2007). Ubiquitinated-protein aggregates form in pancreatic beta-cells during diabetes-induced oxidative stress and are regulated by autophagy. Diabetes.

[205] Quan W., Hur K.Y., Lim Y., Oh S.H., Lee J.C., Kim K.H., Kim G.H., Kim S.W., Kim H.L., Lee M.K. (2012). Autophagy deficiency in beta cells leads to compromised unfolded protein response and progression from obesity to diabetes in mice. Diabetologia.

[206] Fujitani Y., Ebato C., Uchida T., Kawamori R., Watada H. (2009). beta-cell autophagy: A novel mechanism regulating beta-cell function and mass: Lessons from beta-cell-specific Atg7-deficient mice. Islets.

[207] Masini M., Bugliani M., Lupi R., del Guerra S., Boggi U., Filipponi F., Marselli L., Masiello P., Marchetti P. (2009). Autophagy in human type 2 diabetes pancreatic beta cells. Diabetologia.

[208] Zummo F.P., Cullen K.S., Honkanen-Scott M., Shaw J.A.M., Lovat P.E., Arden C. (2017). Glucagon-Like Peptide 1 Protects Pancreatic-Cells From Death by Increasing Autophagic Flux and Restoring Lysosomal Function. Diabetes.

[209] Li X., Zhang L., Meshinchi S., Dias-Leme C., Raffin D., Johnson J.D., Treutelaar M.K., Burant C.F. (2006). Islet microvasculature in islet hyperplasia and failure in a model of type 2 diabetes. Diabetes.

[210] Las G., Serada S.B., Wikstrom J.D., Twig G., Shirihai O.S. (2011). Fatty acids suppress autophagic turnover in beta-cells. J. Biol. Chem..

[211] Dhayal S., Zummo F.P., Anderson M.W., Thomas P., Welters H.J., Arden C., Morgan N.G. (2019). Differential effects of saturated and unsaturated fatty acids on autophagy in pancreatic beta-cells. J. Mol. Endocrinol..

[212] Chu K.Y., O’Reilly L., Mellet N., Meikle P.J., Bartley C., Biden T.J. (2019). Oleate disrupts cAMP signaling, contributing to potent stimulation of pancreatic beta-cell autophagy. J. Biol. Chem..

[213] Wang J., Wu J., Wu H., Liu X.Z., Chen Y.J., Wu J.Y., Hu C.J., Zou D.J. (2015). Liraglutide protects pancreatic beta-cells against free fatty acids in vitro and affects glucolipid metabolism in apolipoprotein E-/- mice by activating autophagy. Mol. Med. Rep..

[214] Hu M., Yang S., Yang L., Cheng Y., Zhang H. (2016). Interleukin-22 Alleviated Palmitate-Induced Endoplasmic Reticulum Stress in INS-1 Cells through Activation of Autophagy. PLoS ONE.

[215] Lin N., Niu Y.X., Zhang W.W., Li X.Y., Yang Z., Su Q. (2017). microRNA-802 is involved in palmitate-induced damage to pancreatic beta cells through repression of sirtuin 6. Int. J. Clin. Exp. Pathol..

[216] Natalicchio A., Tortosa F., Labarbuta R., Biondi G., Marrano N., Carchia E., Leonardini A., Cignarelli A., Bugliani M., Marchetti P. (2015). The p66(Shc) redox adaptor protein is induced by saturated fatty acids and mediates lipotoxicity-induced apoptosis in pancreatic beta cells. Diabetologia.

[217] Wehinger S., Ortiz R., Diaz M.I., Aguirre A., Valenzuela M., Llanos P., Mc Master C., Leyton L., Quest A.F. (2015). Phosphorylation of caveolin-1 on tyrosine-14 induced by ROS enhances palmitate-induced death of beta-pancreatic cells. Biochim. Biophys. Acta.

[218] Xu Q., Chen S.Y., Deng L.D., Feng L.P., Huang L.Z., Yu R.R. (2013). Antioxidant effect of mogrosides against oxidative stress induced by palmitic acid in mouse insulinoma NIT-1 cells. Braz. J. Med. Biol. Res..

[219] Shen X., Yang L., Yan S., Wei W., Liang L., Zheng H., Cai X. (2014). The effect of FFAR1 on pioglitazone-mediated attenuation of palmitic acid-induced oxidative stress and apoptosis in betaTC6 cells. Metabolism.

[220] Cho Y.S., Kim C.H., Kim K.Y., Cheon H.G. (2012). Protective effects of arachidonic acid against palmitic acid-mediated lipotoxicity in HIT-T15 cells. Mol. Cell Biochem..

[221] Lin N., Chen H., Zhang H., Wan X., Su Q. (2012). Mitochondrial reactive oxygen species (ROS) inhibition ameliorates palmitate-induced INS-1 beta cell death. Endocrine.

[222] Lameloise N., Muzzin P., Prentki M., Assimacopoulos-Jeannet F. (2001). Uncoupling protein 2: A possible link between fatty acid excess and impaired glucose-induced insulin secretion?. Diabetes.

[223] Santos L.R., Rebelato E., Graciano M.F., Abdulkader F., Curi R., Carpinelli A.R. (2011). Oleic acid modulates metabolic substrate channeling during glucose-stimulated insulin secretion via NAD(P)H oxidase. Endocrinology.

[224] Koshkin V., Dai F.F., Robson-Doucette C.A., Chan C.B., Wheeler M.B. (2008). Limited mitochondrial permeabilization is an early manifestation of palmitate-induced lipotoxicity in pancreatic beta-cells. J. Biol. Chem..

[225] Redza-Dutordoir M., Averill-Bates D.A. (2016). Activation of apoptosis signalling pathways by reactive oxygen species. Biochim. Biophys. Acta Mol. Cell Res..

[226] Steneberg P., Rubins N., Bartoov-Shifman R., Walker M.D., Edlund H. (2005). The FFA receptor GPR40 links hyperinsulinemia, hepatic steatosis, and impaired glucose homeostasis in mouse. Cell Metab..

[227] Verma M.K., Sadasivuni M.K., Yateesh A.N., Neelima K., Mrudula S., Reddy M., Smitha R., Biswas S., Chandravanshi B., Pallavi P.M. (2014). Activation of GPR40 attenuates chronic inflammation induced impact on pancreatic beta-cells health and function. BMC Cell Biol..

[228] Kung C.P., Murphy M.E. (2016). The role of the p53 tumor suppressor in metabolism and diabetes. J. Endocrinol..

[229] Lovis P., Roggli E., Laybutt R., Gattesco S., Yang J.Y., Widmann C., Abderrahmani A., Regazzi R. (2008). Alterations in microRNA expression contribute to fatty acid-induced pancreatic beta-cell dysfunction. Diabetes.

[230] Zhu Y.X., Sun Y., Zhou Y.C., Zhang Y., Zhang T., Li Y.T., You W.Y., Chang X.A., Yuan L., Han X. (2019). MicroRNA-24 promotes pancreatic beta cells toward dedifferentiation to avoid endoplasmic reticulum stress-induced apoptosis. J. Mol. Cell Biol..

[231] Lin X., Guan H., Huang Z., Liu J., Li H., Wei G., Cao X., Li Y. (2014). Downregulation of Bcl-2 expression by miR-34a mediates palmitate-induced Min6 cells apoptosis. J. Diabetes Res..

[232] Han Y.B., Wang M.N., Li Q., Guo L., Yang Y.M., Li P.J., Wang W., Zhang J.C. (2012). MicroRNA-34a contributes to the protective effects of glucagon-like peptide-1 against lipotoxicity in INS-1 cells. Chin. Med. J..

[233] Li Y., Xu X.J., Liang Y., Liu S.Y., Xiao H.S., Li F., Cheng H., Fu Z.Z. (2010). miR-375 enhances palmitate-induced lipoapoptosis in insulin-secreting NIT-1 cells by repressing myotrophin (V1) protein expression. Int. J. Clin. Exp. Pathol..

[234] Liu Y., Dong J.Y., Ren B. (2018). MicroRNA-182-5p contributes to the protective effects of thrombospondin 1 against lipotoxicity in INS-1 cells. Exp. Med..

[235] Guo R., Yu Y., Zhang Y.J., Li Y.L., Chu X., Lu H.M., Sun C.H. (2020). Overexpression of miR-297b-5p protects against stearic acid-induced pancreatic beta-cell apoptosis by targeting LATS2. Am. J. Physiol. Endocrinol. Metab..

[236] Yu Y., Guo R., Zhang Y.J., Shi H.B., Sun H.R., Chu X., Wu X.Y., Lu H.M., Sun C.H. (2020). miRNA-mRNA profile and regulatory network in stearic acid-treated beta-cell dysfunction. J. Endocrinol..

[237] Chen J., Chen J., Cheng Y., Fu Y., Zhao H., Tang M., Zhao H., Lin N., Shi X., Lei Y. (2020). Mesenchymal stem cell-derived exosomes protect beta cells against hypoxia-induced apoptosis via miR-21 by alleviating ER stress and inhibiting p38 MAPK phosphorylation. Stem Cell Res. Ther..

[238] Noguchi A., Takada M., Nakayama K., Ishikawa T. (2008). cGMP-independent anti-apoptotic effect of nitric oxide on thapsigargin-induced apoptosis in the pancreatic beta-cell line INS-1. Life Sci..

[239] Bachar E., Ariav Y., Cerasi E., Kaiser N., Leibowitz G. (2010). Neuronal nitric oxide synthase protects the pancreatic beta cell from glucolipotoxicity-induced endoplasmic reticulum stress and apoptosis. Diabetologia.

[240] Piro S., Anello M., Di P.C., Lizzio M.N., Patane G., Rabuazzo A.M., Vigneri R., Purrello M., Purrello F. (2002). Chronic exposure to free fatty acids or high glucose induces apoptosis in rat pancreatic islets: Possible role of oxidative stress. Metabolism.

[241] Sargsyan E., Ortsater H., Thorn K., Bergsten P. (2008). Diazoxide-induced beta-cell rest reduces endoplasmic reticulum stress in lipotoxic beta-cells. J. Endocrinol..

[242] Hara T., Mahadevan J., Kanekura K., Hara M., Lu S., Urano F. (2014). Calcium efflux from the endoplasmic reticulum leads to beta-cell death. Endocrinology.

[243] Zhou Y., Sun P., Wang T., Chen K., Zhu W., Wang H. (2015). Inhibition of Calcium Influx Reduces Dysfunction and Apoptosis in Lipotoxic Pancreatic beta-Cells via Regulation of Endoplasmic Reticulum Stress. PLoS ONE.

[244] Vogel J., Yin J., Su L., Wang S.X., Zessis R., Fowler S., Chiu C.H., Wilson A.C., Chen A., Zecri F. (2020). A Phenotypic Screen Identifies Calcium Overload as a Key Mechanism of beta-Cell Glucolipotoxicity. Diabetes.

[245] Rakatzi I., Mueller H., Ritzeler O., Tennagels N., Eckel J. (2004). Adiponectin counteracts cytokine- and fatty acid-induced apoptosis in the pancreatic beta-cell line INS-1. Diabetologia.

[246] Melloul D. (2008). Role of NF-kappaB in beta-cell death. Biochem. Soc. Trans..

[247] Khan S., Kowluru A. (2018). CD36 mediates lipid accumulation in pancreatic beta cells under the duress of glucolipotoxic conditions: Novel roles of lysine deacetylases. Biochem. Biophys. Res. Commun..

[248] Johnson J.D., Han Z., Otani K., Ye H., Zhang Y., Wu H., Horikawa Y., Misler S., Bell G.I., Polonsky K.S. (2004). RyR2 and calpain-10 delineate a novel apoptosis pathway in pancreatic islets. J. Biol. Chem..

[249] Nemcova-Furstova V., Balusikova K., Halada P., Pavlikova N., Sramek J., Kovar J. (2019). Stearate-Induced Apoptosis in Human Pancreatic beta-Cells is Associated with Changes in Membrane Protein Expression and These Changes are Inhibited by Oleate. Proteom. Clin. Appl..

[250] Yamani L., Li B., Larose L. (2015). Nck1 deficiency improves pancreatic beta cell survival to diabetes-relevant stresses by modulating PERK activation and signaling. Cell. Signal..

[251] Chowdhury I., Tharakan B., Bhat G.K. (2008). Caspases—An update. Comp. Biochem. Physiol. B Biochem. Mol. Biol..

[252] Edlich F. (2018). BCL-2 proteins and apoptosis: Recent insights and unknowns. Biochem. Biophys. Res. Commun..

[253] Gu J.Q., Wei Q., Zheng H.Z., Meng X., Zhang J., Wang D.F. (2016). Exendin-4 Promotes Survival of Mouse Pancreatic beta-Cell Line in Lipotoxic Conditions, through the Extracellular Signal-Related Kinase 1/2 Pathway. J. Diabetes Res..

[254] Ahn J.H., Kim M.H., Kwon H.J., Choi S.Y., Kwon H.Y. (2013). Protective Effects of Oleic Acid Against Palmitic Acid-Induced Apoptosis in Pancreatic AR42J Cells and Its Mechanisms. Korean J. Physiol. Pharmacol..

[255] Xiao H.T., Wen B., Ning Z.W., Zhai L.X., Liao C.H., Lin C.Y., Mu H.X., Bian Z.X. (2017). Cyclocarya paliurus tea leaves enhances pancreatic beta cell preservation through inhibition of apoptosis. Sci. Rep..

[256] Cunha D.A., Cito M., Carlsson P.O., Vanderwinden J.M., Molkentin J.D., Bugliani M., Marchetti P., Eizirik D.L., Cnop M. (2016). Thrombospondin 1 protects pancreatic beta-cells from lipotoxicity via the PERK-NRF2 pathway. Cell Death Differ..

[257] Nogueira T.C., Paula F.M., Villate O., Colli M.L., Moura R.F., Cunha D.A., Marselli L., Marchetti P., Cnop M., Julier C. (2013). GLIS3, a Susceptibility Gene for Type 1 and Type 2 Diabetes, Modulates Pancreatic Beta Cell Apoptosis via Regulation of a Splice Variant of the BH3-Only Protein Bim. PLoS Genet..

[258] Tan C., Voss U., Svensson S., Erlinge D., Olde B. (2013). High glucose and free fatty acids induce beta cell apoptosis via autocrine effects of ADP acting on the P2Y(13) receptor. Purinergic Signal..

[259] Grishko V., Rachek L., Musiyenko S., Ledoux S.P., Wilson G.L. (2005). Involvement of mtDNA damage in free fatty acid-induced apoptosis. Free Radic. Biol. Med..

[260] Morishima N., Nakanishi K., Takenouchi H., Shibata T., Yasuhiko Y. (2002). An endoplasmic reticulum stress-specific caspase cascade in apoptosis—Cytochrome c-independent activation of caspase-9 by caspase-12. J. Biol. Chem..

[261] Long J., Su Y.X., Deng H.C. (2014). Lipoapoptosis Pathways in Pancreatic beta-Cells and the Anti-Apoptosis Mechanisms of Adiponectin. Horm. Metab. Res..

[262] Lingohr M. (2003). Decreasing IRS-2 expression in pancreatic β-cells (INS-1) promotes apoptosis, which can be compensated for by introduction of IRS-4 expression. Mol. Cell. Endocrinol..

[263] Marchetti P., Del G.S., Marselli L., Lupi R., Masini M., Pollera M., Bugliani M., Boggi U., Vistoli F., Mosca F. (2004). Pancreatic islets from type 2 diabetic patients have functional defects and increased apoptosis that are ameliorated by metformin. J. Clin. Endocrinol. Metab..

[264] Liadis N., Salmena L., Kwan E., Tajmir P., Schroer S.A., Radziszewska A., Li X., Sheu L., Eweida M., Xu S. (2007). Distinct in vivo roles of caspase-8 in beta-cells in physiological and diabetes models. Diabetes.

[265] Liu H.F., Zhang Z.Y., Li Q. (2017). DR5 but not miRNA-181 or miRNA-211 is involved in ER stress-mediated apoptosis induced by palmitate in islet beta cells. Int. J. Clin. Exp. Pathol..

[266] Bagnati M., Ogunkolade B.W., Marshall C., Tucci C., Hanna K., Jones T.A., Bugliani M., Nedjai B., Caton P.W., Kieswich J. (2016). Glucolipotoxicity initiates pancreatic beta-cell death through TNFR5/CD40-mediated STAT1 and NF-kappa B activation. Cell Death Dis..

[267] Neubauer H., Setiadi P., Gunesdogan B., Pinto A., Borgel J., Mugge A. (2010). Influence of glycaemic control on platelet bound CD40-CD40L system, P-selectin and soluble CD40 ligand in Type 2 diabetes. Diabet. Med..

[268] Cazanave S.C., Mott J.L., Bronk S.F., Werneburg N.W., Fingas C.D., Meng X.W., Finnberg N., El-Deiry W.S., Kaufmann S.H., Gores G.J. (2011). Death Receptor 5 Signaling Promotes Hepatocyte Lipoapoptosis. J. Biol. Chem..

[269] Choi D., Radziszewska A., Schroer S.A., Liadis N., Liu Y., Zhang Y., Lam P.P., Sheu L., Hao Z., Gaisano H.Y. (2009). Deletion of Fas in the pancreatic beta-cells leads to enhanced insulin secretion. Am. J. Physiol. Endocrinol. Metab..

[270] Johnson E.S., Lindblom K.R., Robeson A., Stevens R.D., Ilkayeva O.R., Newgard C.B., Kornbluth S., Andersen J.L. (2013). Metabolomic Profiling Reveals a Role for Caspase-2 in Lipoapoptosis. J. Biol. Chem..

[271] Sheng Q.F., Xiao X.W., Prasadan K., Chen C.D., Ming Y.C., Fusco J., Gangopadhyay N.N., Ricks D., Gittes G.K. (2017). Autophagy protects pancreatic beta cell mass and function in the setting of a high-fat and high-glucose diet. Sci. Rep..

[272] Katsuma S., Hatae N., Yano T., Ruike Y., Kimura M., Hirasawa A., Tsujimoto G. (2005). Free fatty acids inhibit serum deprivation-induced apoptosis through GPR120 in a murine enteroendocrine cell line STC-1. J. Biol. Chem..

